# Radiomics Analysis Based on Contrast-Enhanced MRI for Prediction of Therapeutic Response to Transarterial Chemoembolization in Hepatocellular Carcinoma

**DOI:** 10.3389/fonc.2021.582788

**Published:** 2021-03-31

**Authors:** Ying Zhao, Nan Wang, Jingjun Wu, Qinhe Zhang, Tao Lin, Yu Yao, Zhebin Chen, Man Wang, Liuji Sheng, Jinghong Liu, Qingwei Song, Feng Wang, Xiangbo An, Yan Guo, Xin Li, Tingfan Wu, Ai Lian Liu

**Affiliations:** ^1^Department of Radiology, First Affiliated Hospital, Dalian Medical University, Dalian, China; ^2^Chengdu Institute of Computer Application, Chinese Academy of Sciences, Chengdu, China; ^3^University of Chinese Academy of Sciences, Beijing, China; ^4^Department of Interventional Radiology, First Affiliated Hospital, Dalian Medical University, Dalian, China; ^5^Life Sciences, GE Healthcare, Shanghai, China; ^6^Global Research, GE Healthcare, Shanghai, China; ^7^Clinical Education Team (CET), GE Healthcare, Shanghai, China

**Keywords:** hepatocellular carcinoma, radiomics, magnetic resonance imaging, transarterial chemoembolization, therapeutic response

## Abstract

**Purpose:**

To investigate the role of contrast-enhanced magnetic resonance imaging (CE-MRI) radiomics for pretherapeutic prediction of the response to transarterial chemoembolization (TACE) in patients with hepatocellular carcinoma (HCC).

**Methods:**

One hundred and twenty-two HCC patients (objective response, *n* = 63; non-response, *n* = 59) who received CE-MRI examination before initial TACE were retrospectively recruited and randomly divided into a training cohort (*n* = 85) and a validation cohort (*n* = 37). All HCCs were manually segmented on arterial, venous and delayed phases of CE-MRI, and total 2367 radiomics features were extracted. Radiomics models were constructed based on each phase and their combination using logistic regression algorithm. A clinical-radiological model was built based on independent risk factors identified by univariate and multivariate logistic regression analyses. A combined model incorporating the radiomics score and selected clinical-radiological predictors was constructed, and the combined model was presented as a nomogram. Prediction models were evaluated by receiver operating characteristic curves, calibration curves, and decision curve analysis.

**Results:**

Among all radiomics models, the three-phase radiomics model exhibited better performance in the training cohort with an area under the curve (AUC) of 0.838 (95% confidence interval (CI), 0.753 - 0.922), which was verified in the validation cohort (AUC, 0.833; 95% CI, 0.691 - 0.975). The combined model that integrated the three-phase radiomics score and clinical-radiological risk factors (total bilirubin, tumor shape, and tumor encapsulation) showed excellent calibration and predictive capability in the training and validation cohorts with AUCs of 0.878 (95% CI, 0.806 - 0.950) and 0.833 (95% CI, 0.687 - 0.979), respectively, and showed better predictive ability (*P* = 0.003) compared with the clinical-radiological model (AUC, 0.744; 95% CI, 0.642 - 0.846) in the training cohort. A nomogram based on the combined model achieved good clinical utility in predicting the treatment efficacy of TACE.

**Conclusion:**

CE-MRI radiomics analysis may serve as a promising and noninvasive tool to predict therapeutic response to TACE in HCC, which will facilitate the individualized follow-up and further therapeutic strategies guidance in HCC patients.

## Introduction

Hepatocellular carcinoma (HCC) is the sixth most common malignant tumor worldwide and ranks as the fourth leading cause of cancer-related deaths (1). Curative therapeutic modalities, such as surgical resection, liver transplantation, and local ablative therapy, have been recommended for patients with early-stage HCC (2). Unfortunately, 60% to 70% of HCC patients are already in the intermediate or advanced stage at the time of their first diagnosis, and they can only be treated with palliative treatment (3). Transarterial chemoembolization (TACE) has been accepted as an effective means to control tumor growth, prolong survival, palliate symptoms, and improve quality of life for intermediate stage HCC patients (2, 4). Monitoring tumor response to TACE is a cornerstone in determining therapy efficacy, and plays a critical role in prognosis prediction and future treatment decision-making. Early objective response of HCC to TACE treatment has been identified to be associated with delayed metastasis and better survival (5, 6). Early discrimination of patients with favorable response can facilitate the decision to perform early repeat treatment in order to eradicate remnant viable tumor portions or delay treatment with the aim of decreasing toxicity and treatment-related morbidity. However, patients with HCC who respond poorly to TACE would require timely switching to alternative therapeutic strategies, such as radiofrequency ablation (RFA), resection, or systemic therapy (5, 7, 8).

Several conventional scoring systems that rely on clinical, laboratory, and imaging information have been developed to predict the response to TACE and to guide the decision for retreatment with TACE in HCC patients, including the Assessment for Retreatment with TACE (ART) score (9), the Selection for TACE Treatment (STATE) score (10), and the Hepatoma Arterial Embolization Prognostic (HAP) score (11). However, these scoring systems are not widely used in clinical practice due to their disappointing accuracy (12). Magnetic resonance imaging (MRI) has been regarded as the preferred imaging modality for screening, early detection, and staging in HCC patients, as well as provides imaging biomarkers for prediction of therapeutic response and prognosis (13). Several conventional imaging features have been shown to be associated with negative response of patients with HCC: large tumor size, multiple lesions, irregular margin, faint enhancement on arterial phase, and arterial peritumoral enhancement (7, 14, 15). Although radiologists had attempt to standardize interpretation of liver imaging, the assessment of therapeutic response using such qualitative imaging characteristics remains subjective and variable. In recent years, functional MRI technologies such as diffusion-weighted imaging (DWI), diffusion kurtosis imaging (DKI), and dynamic contrast enhanced MRI (DCE-MRI) have made it possible to effectively and quantitatively evaluate the response of tumors to TACE (13, 16, 17). However, these function imaging techniques require additional acquisitions and stricter scanning conditions and are more affected by respiratory motion, the MR device, scan parameters, etc, which may limit the clinical application and promotion (18).

Radiomics is an emerging method for quantification of tumor heterogeneity by converting images into high-dimensional mineable data (19). The published studies on radiomics of HCC provide encouraging results which have demonstrated the potential utility for prediction of tumor biology, molecular profiles, post-therapy response, and prognosis (20–23). Prior study used computed tomography (CT) - based radiomics analysis to predict therapeutic response to TACE in HCC with a discriminative performance of 0.730 between the responders and non-responders (22). MRI may be also promising in predicting the efficacy of TACE treatment due to the advantage of depicting more soft-tissue characteristics than CT (24). Abajian et al. (25) constructed a model based on clinical data and traditional MR imaging features to predict HCC response to TACE; however, the small population of HCC patients and few imaging features have limited the efficiency and stability of the predictive radiomics model.

In our study, we aimed to investigate the role of contrast-enhanced MRI (CE-MRI) radiomics for predicting the response of HCC to TACE treatment, which may facilitate the individualized follow-up and further therapeutic strategies guidance in HCC patients.

## Materials and Methods

### Patients

**Figure 1:**
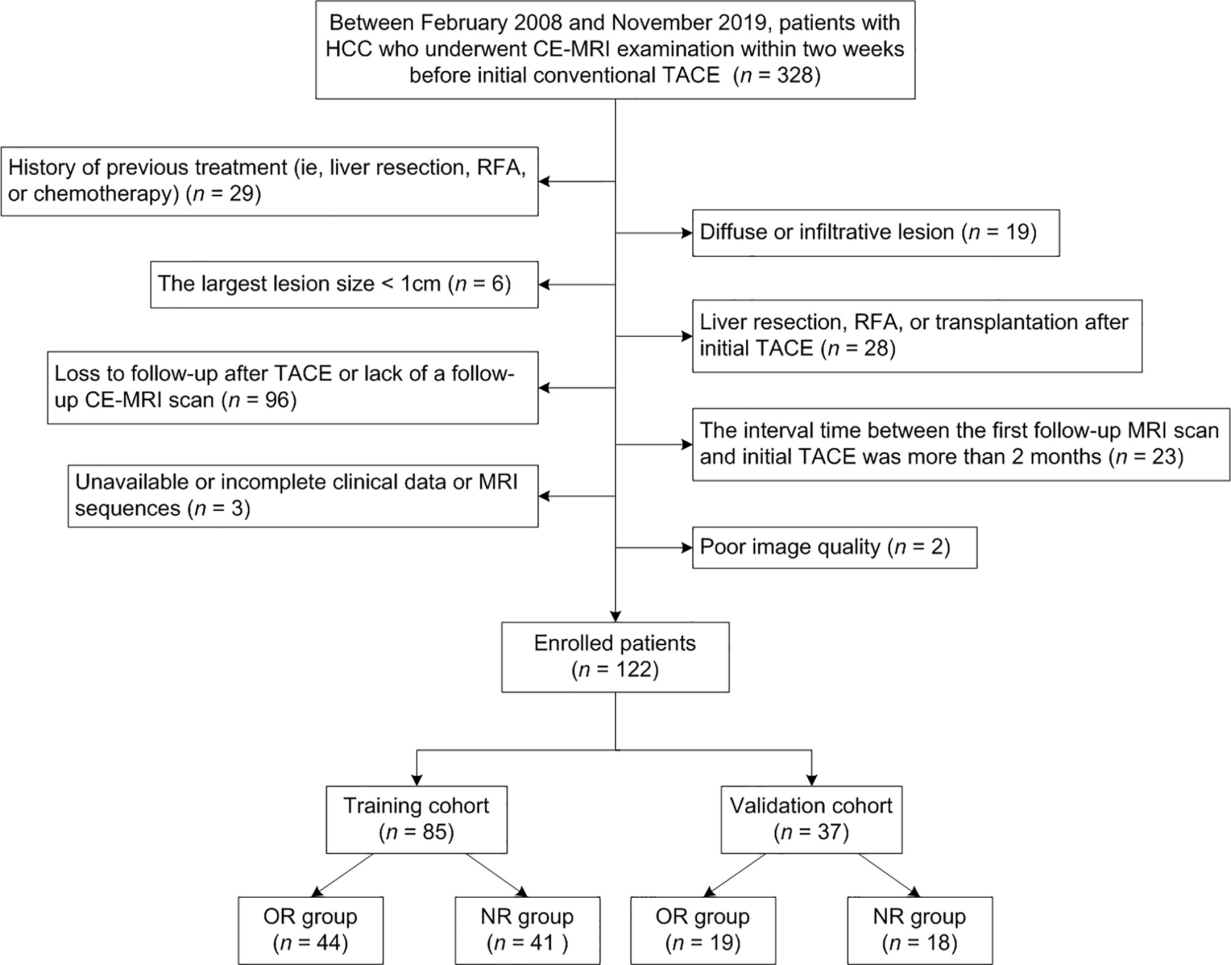
Flowchart of the recruitment pathway for patients.

The ethics committee approved this retrospective study and waived the requirement for informed consent. Between February 2008 and November 2019, 328 consecutive patients with HCC who underwent CE-MRI examination within two weeks before receiving initial conventional TACE at our institution were recruited. The diagnosis of HCC was determined by pathology or imaging features on the basis of the guidelines of the American Association for the Study of Liver Disease (AASLD) (4). However, for the patients who did not meet the noninvasive diagnostic criteria, HCC diagnoses tended to depend on digital subtraction angiography (DSA) examination (23). The exclusion criteria were as follows: (1) previous treatments, including liver resection, RFA, or chemotherapy (*n* = 29); (2) diffuse or infiltrative lesion (*n* = 19); (3) the largest lesion size < 1 cm (*n* = 6); (4) liver resection, RFA, or transplantation after initial TACE (*n* = 28); (5) loss to follow-up after TACE or lack of a follow-up CE-MRI scan (*n* = 96); (6) the interval time between the first follow-up MRI scan and initial TACE was more than 2 months (*n* = 23); (7) unavailable or incomplete clinical data or MRI sequences (*n* = 3); (8) poor image quality (*n* = 2). **Figure 1** illustrates the recruitment pathways for patients. Finally, a total of 122 patients were enrolled and randomly divided into the training cohort (85 cases) and the validation cohort (37 cases) at a ratio of 7:3. The training cohort was used to construct models that were verified by the validation cohort.

Pretherapeutic clinical characteristics, including age, gender, history of hepatitis B or C, alpha-fetoprotein (AFP), alanine aminotransferase (ALT), aspartate aminotransferase (AST), γ-glutamyltranspeptadase (GGT), alkaline phosphatase (ALP), total bilirubin (TBIL), albumin (ALB), platelet count (PLT), prothrombin time (PT), Child-Pugh class, Eastern Cooperative Oncology Group (ECOG) performance status, and Barcelona Clinic Liver Cancer (BCLC) stage, were retrospectively collected.

### MR Data Acquisition

MRI examination was performed using 1.5 T or 3.0 T MR systems (Signa, HDXT, GE Healthcare) with an eight-channel phased array body coil. MR scan sequences included in- and opposed-phase fast-spoiled gradient-recalled echo T1-weighted (T1W) sequence, fat-suppressed fast spin-echo T2-weighted (T2W) sequence, and contrast-enhanced imaging with fat-suppressed T1-weighted three-dimensional (3D) fast-spoiled gradient-recalled echo sequence. The images in arterial phase (AP), portal venous phase (PVP), and delayed phase (DP) were acquired during suspended respiration at 40 s, 70 s, and 90 s, respectively, after initiation of the injection of gadolinium-diethylenetriamine pentaacetic acid (Gd-DTPA) (Bayer Schering Pharma AG) at a patient weight-dependent dose of 0.1 mmol/kg with an injection rate of 2.5 ml/s through median cubital vein. Of the 122 HCC patients described above, 100 patients were examined with the 1.5 T system, and the other 22 patients with the 3.0 T system. The detailed parameters of each scan sequence are listed in **Supplementary Data S1**.

### Analysis of Radiological Features

The imaging features of pretherapeutic MRI were evaluated by two radiologists (reader 1, Y.Z., with 8 years of experience in abdominal MRI; reader 2, N.W., with 7 years of experience in abdominal MRI) in consensus who were aware that the patients had HCC but were blinded to clinical data and imaging report. The radiologists evaluated the following imaging traits: (1) tumor size; (2) tumor location; (3) tumor number (6); (4) tumor shape; (5) tumor margin; (6) intratumor necrosis; (7) intratumor hemorrhage; (8) intratumor fat; (9) tumor encapsulation (26); (10) arterial peritumoral enhancement (27); (11) satellite nodule (23); (12) arterial phase hyperenhancement; (13) washout appearance; (14) liver cirrhosis. If there was any discordance by both radiologists during the imaging analysis, the images were evaluated by another senior radiologist (reader 3, J.H.L., with 20 years of experience in abdominal MRI). Detailed description of imaging features are shown in **Supplementary Data S2**.

### Treatment Modality and Treatment Response Assessment

All conventional TACE procedures were carried out by two interventional radiologists with more than 10 and 5 years of experience with TACE. The detailed description of TACE procedure is shown in **Supplementary Data S3**. Based on pre- and post-therapeutic CE-MRI images, the modified Response Evaluation Criteria in Solid Tumors (mRECIST 1.1) criteria was applied to estimate the tumor response. The mRECIST system classified different responses as follows: complete response (CR), partial response (PR), stable disease (SD), and progression disease (PD) (28). Objective response (OR) referred to sum of CR and PR, whereas non-response (NR) referred to sum of SD and PD (6).

### Tumor Segmentation and Radiomics Feature Extraction

The CE-MRI (AP, PVP, and DP) images exported as digital imaging data and communications in medicine (DICOM) format were loaded into open source software ITK-SNAP (version 3.6.0, http://www.itksnap.org/) for 3D manual segmentation. The region of interests (ROIs) were manually delineated around the entire tumor outline on each axial slice by two abdominal radiologists independently. The ROIs were required to include pseudo-capsule surrounding the tumor and to exclude tumor surrounding vessels. To assess the intra-observer and inter-observer reproducibility, reader 1 performed the segmentation of all patients twice with a 1-month interval and reader 2 independently performed the segmentation of all patients followed the same procedure. The reproducibility was analyzed by calculating intraclass correlation coefficient (ICC).

**Figure 2:**
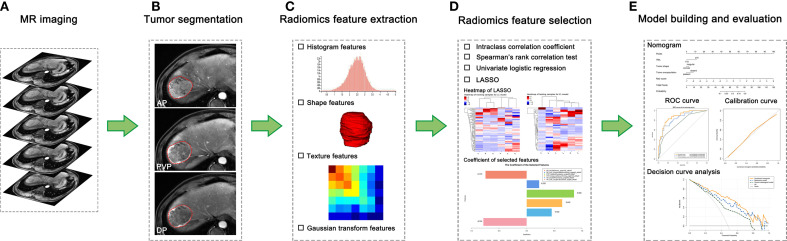
The workflow of radiomics analysis in our study. **(A)** Contrast-enhanced MR imaging was acquired. **(B)** Tumors were manually delineated around the entire tumor outline on all axial slices of arterial phase (AP), portal venous phase (PVP), and delayed phase (DP) images, and three-dimensional segmentations were formed. **(C)** Total 2367 radiomics features were extracted. **(D)** Four steps of feature dimensionality reduction were applied to all extracted features. **(E)** The radiomics model was constructed using logistic regression algorithm, and a nomogram that incorporates the radiomics score and clinical-radiological risk factors was established to provide a more understandable treatment response measurement for individualized evaluation, followed by receiver operating characteristic curve analysis, calibration curve, and decision curve analysis.

Image preprocessing and feature extraction were performed using A. K. software (Artificial Intelligence Kit, Version 3.2.5, GE Healthcare). Images were resampled to a voxel size of 1 × 1 × 1 mm *via* linear interpolation algorithm, which could correct the pixel-spacing difference and restore the tumor volume, allowing for a constant intensity resolution across all tumor images (21, 29, 30). Normalization of signal intensity was performed to correct the scanner effect because MRI signal intensity is usually relative with large differences between scanners (29, 31). Next, 789 radiomics features from each enhanced phase were extracted, including the following categories: 42 histogram features, 144 gray level co-occurrence matrix (GLCM), 180 gray level run length matrix (GLRLM), 11 grey-level zone size matrix (GLZSM), 10 Haralick features, 15 form factors, and 387 Gaussian transform features. A total of 2367 features were extracted when all three phases were used. Details of radiomics features are listed in **Supplementary Data S4**. Values of extracted radiomics features were standardized using z-score in the training cohort, and the feature values of the validation cohort were then z-score standardized by using the mean and standard deviation values of each radiomics feature derived from the training cohort (30, 31). The workflow of the radiomics analysis is depicted in **Figure 2**.

### Feature Selection and Radiomics Model Construction

A four-step procedure was devised for dimensionality reduction. First, in order to ensure the robust and reproducibility of the model, the radiomics features with high stability in both intra-observer and inter-observer (ICC values > 0.8) were selected for subsequent analysis. Second, the Spearman’s rank correlation test was applied to exclude the redundant features (correlation coefficient values ≥ 0.9). Next, the features with significant differences between the OR and NR groups were selected using univariate logistic regression (*P* < 0.05). Finally, the least absolute shrinkage and selection operator (LASSO) logistic regression algorithm, with penalty parameter tuning conducted by 5-fold cross-validation, was further performed to identify the top-ranked and most valuable features to build the predictive model. The radiomics models were constructed using the selected features based on each phase and their combination (AP, PVP, DP, AP-PVP, AP-DP, PVP-DP, and AP-PVP-DP) *via* the multivariate logistic regression analysis. The radiomics score (rad-score) was calculated for each patient *via* a linear combination of the selected radiomics features weighted by their respective coefficients.

### Clinical-Radiological Model Construction

Variables with *P* value < 0.1 in the univariate analysis were included in the multivariate logistic regression analysis to identify the independent clinical-radiological risk factors associated with therapeutic response (*P* < 0.05). Odds ratio and 95% confidence interval (CI) were calculated for each risk factor. The clinical-radiological model was constructed using the above independent risk factors *via* multivariate logistic regression algorithm.

### Combined Model Construction and Nomogram Development

A combined model, which incorporated the radiomics score derived from the highest performance radiomics model and the independent clinical-radiological risk factors for predicting tumor response, was established based on the proposed logistic regression analysis. The collinearity analysis of the radiomics score and the clinical-radiological risk factors was assessed using variance inflation factor (VIF) (32). A nomogram was then constructed based on the combined model to provide a visual tool for clinical usefulness. In addition, we have constructed a radiological-radiomics model which integrated the independent radiological predictors and the radiomics score (based on the highest performance radiomics model) for tumor response prediction.

### Statistical Analysis

Continuous variables among clinical-radiological characteristics in the training and validation cohorts were compared using the Student’s *t*-test or Mann-Whitney *U*-test, and categorical variables were compared using the chi-squared test or Fisher’s exact test, when appropriate. The discrimination performance was evaluated by using receiver operating characteristic (ROC) curves in each model. The area under the curves (AUCs) of the ROC curves, as well as accuracy, sensitivity, and specificity were obtained. Comparisons between the AUCs of various models were performed using the Delong’s test. We also performed stratified analysis on the subgroups of MRI scanner of our radiomics models. We used the ROC curve and AUC to evaluate the performance of prediction models on the subpopulations. Calibration curves were plotted to evaluate the predictive accuracy of the nomogram, accompanied by the Hosmer - Lemeshow test, and *P* values > 0.05 were considered good. Decision curve analysis (DCA) was conducted to estimate the clinical utility based on the net benefit of the radiomics model, clinical-radiological model, and nomogram across different threshold probabilities. All statistical analyses were conducted with R software (version 3.6.1, http://www.R-project.org). A two-sided *P* value < 0.05 was considered statistically significant.

## Results

### Patient Characteristics

A total of 122 patients with HCC (109 male, 13 female; median age, 59 years; range, 44 - 83 years) were ultimately collected in the study. The diagnosis of HCC was determined by pathology in 10 patients and by specific imaging features on the basis of the AASLD guidelines in 112 patients. The median interval time between the initial TACE and the first post-therapeutic MRI was 38 days (range, 25 - 59 days). On the basis of the mRECIST criteria, the patients for CR, PR, SD, and PD were 27 (22.1%), 36 (29.5%), 49 (40.2%), and 10 (8.2%), respectively. The baseline characteristics in the training and validation cohorts are summarized in **Table 1**. No significant difference was observed in the demographic data, clinical characteristics, or radiological features, except for ECOG performance status between the training and validation cohorts.

**Table 1:** Patient baseline characteristics.

Variables	Training cohort (*n* = 85)	Validation cohort (*n* = 37)	*Z/F* value	*P* value
Patient demographics				
Age (years), median (interquartile range)	59 (56, 65)	60 (54, 65)	-0.136	0.891
Gender, No. (%)			0.127	0.722
Male	77 (90.6)	32 (86.5)		
Female	8 (9.4)	5 (13.5)		
Clinical characteristics				
History of hepatitis B or C, No. (%)			0.012	0.913
Positive	56 (65.9)	24 (64.9)		
Negative	29 (34.1)	13 (35.1)		
AFP (IU/ml), No. (%)			0.072	0.788
≤ 400	55 (64.7)	23 (62.2)		
> 400	30 (35.3)	14 (37.8)		
ALT (U/L), No. (%)			3.376	0.066
≤ 50	47 (55.3)	27 (73.0)		
> 50	38 (44.7)	10 (27.0)		
AST (U/L), No. (%)			3.751	0.053
≤ 40	30 (35.3)	20 (54.1)		
> 40	55 (64.7)	17 (45.9)		
GGT (U/L), No. (%)			3.107	0.078
≤ 60	21 (24.7)	15 (40.5)		
> 60	64 (75.3)	22 (59.5)		
ALP (U/L), No. (%)			0.004	0.948
≤ 125	50 (58.8)	22 (59.5)		
> 125	35 (41.2)	15 (40.5)		
TBIL (umol/L), No. (%)			0.001	0.977
≤ 19	48 (56.5)	21 (56.8)		
> 19	37 (43.5)	16 (43.2)		
ALB (g/L), No. (%)			0.072	0.788
< 40	55 (64.7)	23 (62.2)		
≥ 40	30 (35.3)	14 (37.8)		
PLT (×10^9^/L), No. (%)			2.864	0.091
< 125	44 (51.8)	13 (35.1)		
≥ 125	41 (48.2)	24 (64.9)		
PT (s), No. (%)			0.050	0.822
≤ 13	51 (60.0)	23 (62.2)		
> 13	34 (40.0)	14 (37.8)		
Child-Pugh class, No. (%)			0.009	0.924
A	65 (76.5)	28 (75.7)		
B	20 (23.5)	9 (24.3)		
ECOG performance status, No. (%)			4.500	0.034
0	77 (90.6)	28 (75.7)		
1	5 (5.9)	7 (18.9)		
2	3 (3.5)	2 (5.4)		
BCLC stage, No. (%)			2.120	0.145
A	41 (48.2)	15 (40.6)		
B	31 (36.5)	10 (27.0)		
C	13 (15.3)	12 (32.4)		
Radiological features				
Tumor size, No. (%)			2.064	0.151
≤ 5 cm	51 (60.0)	17 (45.9)		
> 5 cm	34 (40.0)	20 (54.1)		
Tumor location, No. (%)			1.766	0.184
Left lobe	20 (23.5)	6 (16.2)		
Junction lobe	1 (1.2)	2 (5.4)		
Right lobe	63 (74.1)	28 (75.7)		
Caudate lobe	1 (1.2)	1 (2.7)		
Tumor number, No. (%)			2.349	0.125
≤ 3	77 (90.6)	37 (100.0)		
> 3	8 (9.4)	0 (0.0)		
Tumor shape, No. (%)			1.761	0.184
Circular	67 (78.8)	25 (67.6)		
Irregular	18 (21.2)	12 (32.4)		
Tumor margin, No. (%)			0.780	0.377
Smooth	64 (75.3)	25 (67.6)		
Non-smooth	21 (24.7)	12 (32.4)		
Intratumor necrosis, No. (%)			0.364	0.546
Present	23 (27.1)	12 (32.4)		
Absent	62 (72.9)	25 (67.6)		
Intratumor hemorrhage, No. (%)			0.009	0.924
Present	20 (23.5)	9 (24.3)		
Absent	65 (76.5)	28 (75.7)		
Intratumor fat, No. (%)			1.059	0.303
Present	12 (14.1)	8 (21.6)		
Absent	73 (85.9)	29 (78.4)		
Tumor encapsulation, No. (%)			1.290	0.256
Present	53 (62.4)	19 (51.4)		
Absent	32 (37.6)	18 (48.6)		
Arterial peritumoral enhancement, No. (%)			1.395	0.238
Present	21 (24.7)	13 (35.1)		
Absent	64 (75.3)	24 (64.9)		
Satellite nodule, No. (%)			0.013	0.910
Present	8 (9.4)	3 (8.1)		
Absent	77 (90.6)	34 (91.9)		
Arterial phase hyperenhancement, No. (%)			1.445	0.229
Present	79 (92.9)	37 (100.0)		
Absent	6 (7.1)	0 (0.0)		
Washout appearance, No. (%)			0.133	0.715
Present	58 (68.2)	24 (64.9)		
Absent	27 (31.8)	13 (35.1)		
Liver cirrhosis, No. (%)			1.536	0.215
Present	56 (65.9)	20 (54.1)		
Absent	29 (34.1)	17 (45.9)		

Except where indicated, data are shown as numbers of patients, with percentages in parentheses. No significant difference was found between the training and validation cohorts, except for ECOG performance status. AFP, alpha-fetoprotein; ALT, alanine aminotransferase; AST, aspartate aminotransferase; GGT, γ-glutamyltranspeptadase; ALP, alkaline phosphatase; TBIL, total bilirubin; ALB, albumin; PLT, platelet count; PT, prothrombin time; ECOG, Eastern Cooperative Oncology Group; BCLC, Barcelona Clinic Liver Cancer.

### Feature Selection and Radiomics Model Building

After ICC analysis, a total of 1545 radiomics features were considered stable with both intra-observer and inter-observer stability (474 features from AP, 523 features from PVP, and 548 features from DP; ICC range: 0.804 - 0.999, 0.802 - 0.995, 0.802 - 0.995, respectively). These features obtained by reader 1 in the first measurement were used for subsequent data analysis. The Spearman’s rank correlation test, univariate logistic regression, and LASSO logistic regression were then used for dimensionality reduction in order. Based on each phase and their combination (AP, PVP, DP, AP-PVP, AP-DP, PVP-DP, and AP-PVP-DP), the 7, 6, 10, 6, 5, 7, and 6 radiomics features were ultimately selected, respectively, applying for the radiomics model building. The formulae of calculating the rad-score for each patient are described in **Supplementary Data S5**.

### Clinical-Radiological Model Building

The univariate and multivariate logistic regression analyses for the prediction of therapeutic response in the training cohort are shown in **Supplementary Data S6**. In the univariate analysis, 1 clinical characteristic (TBIL) and 5 radiological features (tumor size, tumor shape, tumor encapsulation, satellite nodule, and washout appearance) were found significantly different between the OR and NR groups (all *P* < 0.05). Multivariate analysis indicated that TBIL (Odd ratio = 0.342; 95% CI: 0.130 - 0.904; *P* = 0.031), tumor shape (Odd ratio = 4.468; 95% CI: 1.216 - 16.415; *P* = 0.024), and tumor encapsulation (Odd ratio = 0.354; 95% CI: 0.130 - 0.964; *P* = 0.042) were independent risk factors for predicting therapeutic response. The above three factors were applied for clinical-radiological model construction.

### Combined Model Building, Nomogram Construction and Model Evaluation

**Figure 3:**
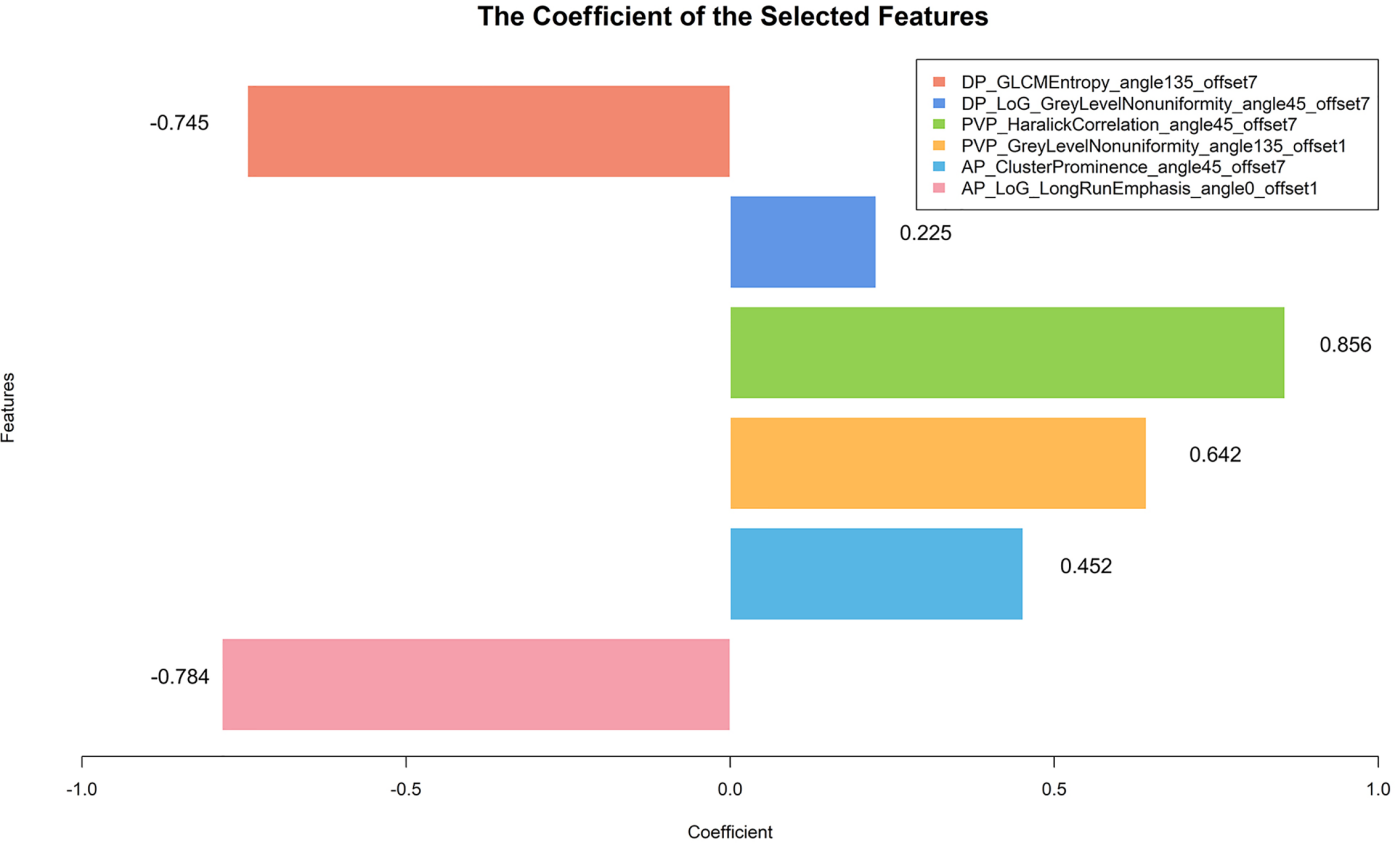
The histogram exhibits radiomics features contributed to the constructed radiomics model based on three-phase images. The y-axis represents radiomics features, with their coefficients in the multivariate logistic regression analysis plotted on the x-axis.

**Figure 4:**
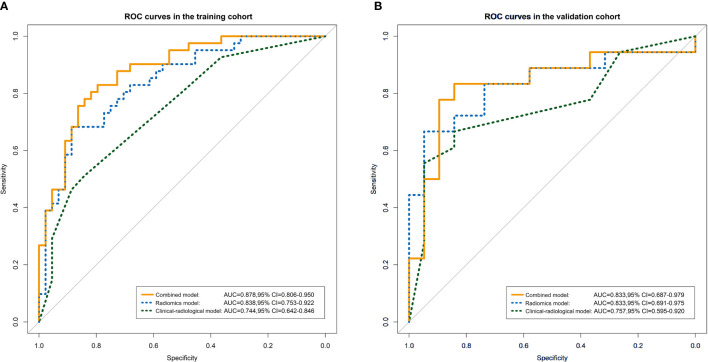
ROC curves for the radiomics model, clinical-radiological model, and combined model in the training cohort **(A)** and validation cohort **(B)**.

We built 10 predictive models, including 1 clinical-radiological model, 7 radiomics models, 1 combined model, and 1 radiological-radiomics model. The clinical-radiological model yielded AUCs of 0.744 (95% CI, 0.642 - 0.846) and 0.757 (95% CI, 0.595 - 0.920) in the training and validation cohorts. Among all radiomics models, the three-phase radiomics model (AP-PVP-DP model) had better discrimination capacity between the OR and NR groups, with AUCs of 0.838 (95% CI, 0.753 - 0.922) and 0.833 (95% CI, 0.691 - 0.975) in the training and validation cohorts. The AP-PVP-DP model individual feature coefficients are presented in **Figure 3**. For the analysis of radiomics models of the single phase, PVP model showed higher AUCs of 0.797 (95% CI, 0.705 - 0.890) and 0.830 (95% CI, 0.684 - 0.977) in the two cohorts. In addition, the stratified analysis showed that our radiomics models were not influenced by MRI scanners with different magnetic field strength (all *P* > 0.05) (shown in **Supplementary Data S7**). The combined model integrating clinical-radiological factors (TBIL, tumor shape, and tumor encapsulation) and radiomics score (based on AP-PVP-DP model) was constructed, showing preferable predictive performance with AUCs of 0.878 (95% CI, 0.806 - 0.950) and 0.833 (95% CI, 0.687 - 0.979) in the training and validation cohorts. The VIFs of four potential predictors (TBIL, tumor shape, tumor encapsulation, and radiomics score) ranged from 1.007 to 1.219, which indicated that those predictors were not so highly correlated. The combined model demonstrated a significantly higher AUC than the clinical-radiological model in the training cohort (*P* = 0.003), whereas there was no significant difference in the AUC between the two models in the validation cohort (*P* = 0.239). No significant differences in AUC values were found between the combined model and the radiomics model (*P* = 0.155, 1.000) and between the clinical-radiological model and the radiomics model (*P* = 0.148, 0.344), respectively, in the training and validation cohorts. The discriminative performance of different predictive models are shown in **Table 2**, **Figures 4A, B**. Performance evaluation of the radiological-radiomics model is shown in **Supplementary Data S8**.

**Table 2:** Discriminative performance of different predictive models in the training and validation cohorts.

Predictive models	Training cohort				Validation cohort			
	AUC (95% CI)	Accuracy	Sensitivity	Specificity	AUC (95% CI)	Accuracy	Sensitivity	Specificity
Clinical-radiological model	0.744 (0.642 - 0.846)	0.682	0.512	0.841	0.757 (0.595 - 0.920)	0.757	0.667	0.842
Radiomics model								
AP	0.774 (0.675 - 0.873)	0.682	0.659	0.705	0.752 (0.592 - 0.911)	0.649	0.667	0.632
PVP	0.797 (0.705 - 0.890)	0.682	0.610	0.750	0.830 (0.684 - 0.977)	0.784	0.833	0.737
DP	0.736 (0.629 - 0.843)	0.682	0.561	0.795	0.757 (0.592 - 0.923)	0.730	0.667	0.789
AP-PVP	0.818 (0.729 - 0.907)	0.718	0.683	0.750	0.810 (0.671 - 0.949)	0.757	0.667	0.842
AP-DP	0.780 (0.681 - 0.879)	0.718	0.732	0.705	0.804 (0.652 - 0.956)	0.703	0.833	0.579
PVP-DP	0.800 (0.707 - 0.893)	0.706	0.683	0.727	0.830 (0.690 - 0.971)	0.757	0.889	0.632
AP-PVP-DP	0.838 (0.753 - 0.922)	0.753	0.732	0.773	0.833 (0.691 - 0.975)	0.703	0.889	0.526
Combined model	0.878 (0.806 - 0.950)	0.812	0.805	0.818	0.833 (0.687 - 0.979)	0.730	0.833	0.632

AP, arterial phase; PVP, portal venous phase; DP, delayed phase; AUC, area under the curve; CI, confidence interval.

**Figure 5:**
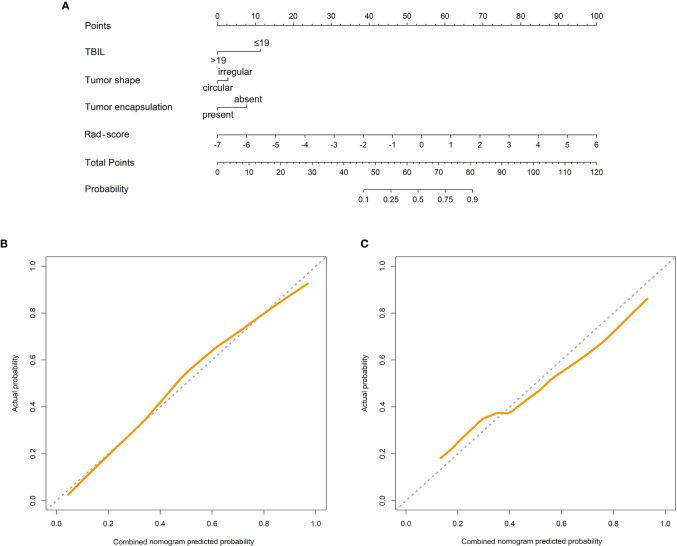
Combined nomogram **(A)**. The combined nomogram incorporated total bilirubin (TBIL), tumor shape, tumor encapsulation, and the radiomics score (rad-score). Calibration curves of the combined nomogram in the training cohort **(B)** and the validation cohort **(C)**. The y-axis represents the actual result, and the x-axis represents the predicted probability. The diagonal dashed line indicates the ideal prediction by a perfect model. The solid line indicates the predictive performance of the model. If the solid line is closer to the diagonal dashed line, it means a better prediction.

**Figure 6:**
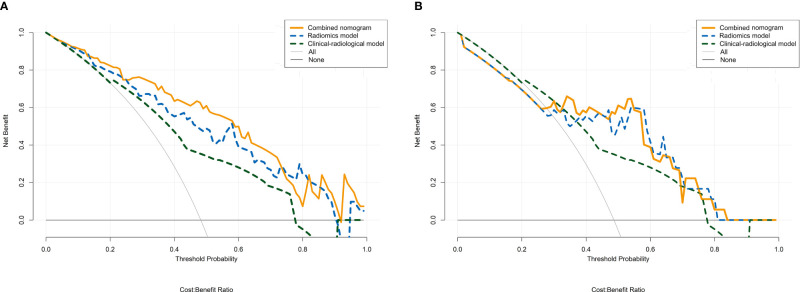
Decision curve analysis for the radiomics model, clinical-radiological model, and combined nomogram in the training cohort **(A)** and the validation cohort **(B)**. The y-axis represents the net benefit, and the x-axis represents the threshold probability. The radiomics model, clinical-radiological model, and combined nomogram obtained more benefit than either the treat-all-patients scheme (gray line) or the treat-none scheme (horizontal black line) within certain ranges of threshold probabilities for predicting therapeutic response to TACE in HCC.

The combined nomogram was established based on the combined model to individually predict tumor response of HCC patients after TACE treatment (**Figure 5A**). Furthermore, the calibration curves demonstrated favorable calibration of the combined nomogram in the training and validation cohorts (**Figures 5B, C**). The Hosmer-Lemeshow test yielded non-significant results in the two cohorts (all *P* > 0.05), suggesting a satisfying fit of the nomogram. The decision curves displayed good performance of the radiomics model, clinical-radiological model, and combined nomogram in terms of clinical utility, which added more benefit than either the treat-all or treat-none scheme across the majority of the range of reasonable threshold probabilities in the training and validation cohorts (**Figures 6A, B**).

## Discussion

In the present study, we established a novel radiomics-based nomogram incorporating the clinical-radiological characteristics and radiomics score from pretherapeutic CE-MRI images to predict therapeutic response to TACE in HCC patients. Our nomogram showed a satisfactory performance with AUCs of 0.878 and 0.833, respectively, in the training and validation cohorts. To the best of our knowledge, this is the first study to develop a radiomics model, using radiomics features from MRI to predict therapeutic response of HCC undergoing TACE so far. The proposed radiomics approach may aid in assessment of the efficacy of TACE and facilitate prognosis prediction and further treatment planning for unresectable HCC patients.

In our study, most of HCC patients receiving TACE therapy had BCLC A or B stage, which was in accordance with previous studies (23, 33). TACE is the standard therapy that recommended for HCC patients with BCLC stage B. For patients with BCLC A stage, TACE is not ideal therapeutic modality, but could be an alternative option for those patients for whom ablation or resection would be unsuitable due to several factors, such as age, severe complications, and tumor location (23, 33). Therefore, HCC patients included in our study reflect the real phenomenon in clinical setting.

Previous studies have showed that early response assessment at initial TACE session is a significant and robust prognostic indicator, which may help the modification of further treatment strategies in an optimized manner in clinical practice (6, 34, 35). Several scholars have focused on therapeutic response assessment to TACE using texture analysis based on CT or MRI images (36, 37). Park et al. (36) investigated texture analysis based on hepatic-arterial CT images in 132 HCCs treated with TACE, showing that tumors in CR group have significantly lower homogeneity and higher mean attenuation, GLCM moments, and CT number percentiles, and these parameters would be helpful in prediction of therapeutic response before the implementation of TACE. A study of 89 HCC patients has also identified the value of texture analysis based on enhanced MRI for predicting an early therapeutic response to TACE combined with high-intensity focused ultrasound treatment in HCC patients (37). However, the predictive efficiencies in above studies were limited (with the highest AUCs of 0.720 and 0.760, respectively).

Radiomics focuses on improvement of image analysis by extracting large amounts of quantitative features through different mathematical algorithms and would be expected to improve the diagnostic performance *via* medical images (38). In the current study, the radiomics features were extracted from AP, PVP, and DP images of CE-MRI to build predictive radiomics models. The radiomics model based on PVP images showed a superior predictive performance compared to those based on AP or DP images. This indicated that pretherapeutic PVP images could capture more information to reflect the heterogeneity of tumors, which was consistent with previous studies (23, 39). Among all radiomics models, the AP-PVP-DP radiomics model showed better discriminative power between the OR and NR groups. We suggested that three-phase CE-MRI contained more potential tumor heterogeneous information, and the multiparametric approach was required for post-therapeutic prognostic analysis of HCC (31, 40). In our AP-PVP-DP model, six most important radiomics features for predicting non-response were as follows: AP_ClusterProminence_angle45_offset7, AP_LoG_LongRunEmphasis_angle0_offset1, PVP_HaralickCorrelation_angle45_offset7, PVP_GreyLevelNonuniformity_angle135_offset1, DP_GLCMEntropy_angle135_offset7, and DP_LoG_GreyLevelNonuniformity_angle45_offset7. The above features describe the patterns or spatial distribution of voxel intensities within the ROI, and they have served as recognized parameters to capture tumor heterogeneity. Intratumor heterogeneity can be caused by variations in cellularity, angiogenesis, extracellular matrix, or necrosis and therefore has the potential to be an important prognostic factor (41). Recently, there has been an increasing interest in tumor heterogeneity quantification and its effect on treatment responses. Several studies have demonstrated that the tumor response is closely related to tumor heterogeneity identified by imaging radiomics features (22, 36, 37). A recent study by Morshid et al. (22) suggested that CT-based radiomics had moderate predictive performance with the AUC of 0.733 for predicting the response to TACE in 105 HCC patients. Our AP-PVP-DP model displayed a better performance than the previous study, which may due to the following advantages: first, MR imaging can provide better contrast and resolution in soft-tissue; and second, we analyzed three-phase enhanced images, which were possess of more image features and may more fully reflect tumor heterogeneity. In addition, it is noted that the imaging data resulted from the use of two MRI scanners with different magnetic field strength might effect the variability of MRI signal intensity with a resultant bias in the assessment of radiomics features. However, normalization of signal intensity was performed before tumor segmentation and thus to correct the scanner effect. This phenomenon might also reflect clinical reality because a mixture use of MRI scanners occurs frequently (23). In this study, the stratified analysis showed that our radiomics models were not influenced by MRI scanners with different magnetic field strength, which indicating good generalizability and robustness of the prediction models.

In our study, clinical-radiological factors including TBIL, tumor shape and tumor encapsulation were used to construct the predictive model. We suggested that the TBIL was an independent risk factor for tumor response. However, several previous studies have demonstrated that the TBIL cannot be used as a clinical risk factor for the estimation of treatment efficacy but could predict survival in HCC patients treated with TACE (15, 25). Further research should be conducted on the correlation between the TBIL and treatment efficacy of TACE. Our study indicated that tumors with irregular shape were inclined to show non-response to TACE. This might be interpreted that the irregular morphology represent more active growth pattern and more aggressive biological behavior. Our study demonstrated that tumors without encapsulation showed poorer response in HCC patients undergoing TACE, which was similar to previous studies (31, 42). The absence of the tumor encapsulation has been reported to be one of imaging features of microvascular invasion in HCC, and it may have correlation with more strongly aggressiveness and poorer survival (30, 31, 42, 43). Nevertheless, our clinical-radiological model merely showed moderate predictive performance with AUCs of 0.744 and 0.757 separately in the training and validation cohorts, which may indicate that basic clinical and imaging traits are rough surrogates for tumor biology.

Furthermore, adding the radiomics score to the clinical-radiological model can lead to significant improvement of the predictive efficiency in the training cohort (AUC, 0.744 to 0.878, *P* = 0.003), which indicates that the multimarker analysis combining the MRI radiomics features and clinical-radiological characteristics maximizes the predictive value of therapy effectiveness, and may potentially provide additional valuable information about tumor biology and heterogeneity. Interestingly, the radiomics model alone performed well in predicting the treatment efficacy compared with the combined model (*P* > 0.05). We speculated the reason that conventional clinical-radiological factors losing its association in the combined model may be that these clinical-radiological factors have much less impact on the model than radiomics model (32). In additional, the combined model had no significant improvement in the AUC compared to the clinical-radiological model in the validation cohort (*P* = 0.239), but it was a trend that when adding the radiomics score, the performance was better than the single clinical-radiological model with higher AUC. We speculated the phenomenon was correlated with a small sample size of HCC patients, and such application will require further study for verification. Finally, this study also constructed an easy-to-use, graphical analog computation device—the nomogram, which allows clinicians to obtain results quickly and reliably by simply drawing several lines (44). The nomogram based on the combined model showed good discrimination, calibration, and clinical usefulness, which carries potential clinical significance in assisting clinicians for the visual and personalized estimation of treatment efficacy of TACE in HCC patients.

There were several limitations in our study. First, it was a single-center study without external validation. Second, the retrospective nature of the study, the small population as well as the long duration of the inclusion period, may affect the robustness of our conclusions. Thus, a larger cohort population of the prospective study based on multi-center is necessary in the future to verify the performance of proposed predictive models. Third, most of patients in our study were not confirmed by pathology. In the future, we will try to collect more HCC patients with pathological results to reinforce the conclusions of our study. Fourth, the segmentation of entire tumor was manually delineated by radiologists, and thus is time-consuming and labor intensive and prone to user variability. In the future, we will try to develop an automatic and reliable liver tumor segmentation method to solve the problem. In addition, it should be noted that the multi-sequence MRI data are not included in this study. In the future, we will attempt to develop a radiomics approach based on multi-sequence MRI for response evaluation in HCC patients after TACE treatment. Finally, future efforts are still necessary to discuss MRI-based deep learning model for predicting the response to TACE with the hope to improve the predictive ability.

In conclusion, radiomics features based on pretherapeutic CE-MRI images may be potential biomarkers for predicting HCC response to TACE. The combined nomogram integrating the radiomics score with clinical-radiological risk factors demonstrates a favorable discrimination performance, and may aid in the individualized and visualized prediction of therapeutic response of HCC patients undergoing TACE. The proposed methodology may facilitate clinical decision-making and could potentially recognize patients who would benefit from TACE, thereby further guide treatment planning.

## Data Availability Statement

The raw data supporting the conclusions of this article will be made available by the authors, without undue reservation.

## Ethics Statement

The studies involving human participants were reviewed and approved by The First Affiliated Hospital of Dalian Medical University. Written informed consent for participation was not required for this study in accordance with the national legislation and the institutional requirements.

## Author Contributions

Guarantor of the article: AL. Conception and design: AL, YZ, NW, and JW. Collection and assembly of data: YZ, NW, QZ, TL, MW, LS, JL, QS, FW, and XA. Data analysis and interpretation: YZ, NW, YY, ZC, JL, YG, XL, and TW. Manuscript writing: YZ and NW. Manuscript editing: AL, JW, and YG. All authors contributed to the article and approved the submitted version.

## Funding

This work was supported by the National Natural Science Foundation of China (grant number 61971091) and the Program for Training Capital Science and Technology Leading Talents (grant number Z181100006318003). The funding body contributes to the design of the study and analysis of data.

## Conflict of Interest

The authors declare that the research was conducted in the absence of any commercial or financial relationships that could be construed as a potential conflict of interest.
