# Supplementary material for: Radiomics Analysis Based on Contrast-Enhanced MRI for Prediction of Therapeutic Response to Transarterial Chemoembolization in Hepatocellular Carcinoma
Source: Front Oncol. 2021 Mar 31;11:582788. doi: 10.3389/fonc.2021.582788 (PMC8045706; doi:10.3389/fonc.2021.582788)
Supplement: Supplementary file 1 [file DataSheet_1.doc]

**Supplementary Data**

S1. Magnetic resonance imaging sequences and parameters

S2. Detailed description of imaging features

S3. Detailed description of TACE procedure

S4. Detailed name and description of radiomics features

4.1 Histogram features

4.2 Grey level co-occurrence matrix (GLCM) features

4.3 Grey level run length matrix (GLRLM) features

4.4 Grey-level zone size matrix (GLZSM) features

4.5 Haralick features

4.6 Form factors

4.7 Gaussian transform features

S5. The formulae of the rad-score in each phase and their combination

S6. Univariate and multivariate analyses of clinical-radiological characteristics between objective response and non-response groups in the training cohort

S7. Stratified prediction performance on the subgroups of MRI scanner

S8. Performance evaluation of the radiological-radiomics model

S9. References

**S1. Magnetic resonance imaging sequences and parameters**

| Acquisition sequences | Field strength | TR (ms) | TE (ms) | FOV (mm^2^) | Matrix size | NEX | Section thickness (mm) | Fat suppression |
| --- | --- | --- | --- | --- | --- | --- | --- | --- |
| T1-weighted IP and OP imaging | 1.5T | 5.8 | 4.2 and 2.1 | 400 × 360 | 256 × 170 | 0.70 | 5 | No |
|  | 3.0T | 4 | 2.5 and 1.2 | 400 × 360 | 288 × 224 | 0.69 | 5 | No |
| T2-weighted FSE | 1.5T | 6667 | 92.6 | 440 × 330 | 256 × 256 | 2.00 | 6.5 | Yes |
|  | 3.0T | 7059 | 96.1 | 440 × 396 | 288 × 224 | 2.00 | 6 | Yes |
| Contrast-enhanced imaging with T1-weighted 3D GRE | 1.5T | 5.8 | 3.1 | 400 × 360 | 256 × 170 | 0.70 | 5 | Yes |
|  | 3.0T | 4 | 1.8 | 420 × 378 | 270 × 224 | 0.70 | 5 | Yes |

**Note:** *IP*, in-phase; *OP*, opposed-phase; *FSE*, fast spin-echo; *3D*, three-dimensional; *GRE*, gradient-recalled echo; *TR*, repetition time; *TE*, echo time; *FOV*, field of view; *NEX*, number of excitation.

**S2. Detailed description of imaging features**

The radiologists evaluated the following imaging traits: (1) tumor size (the largest axial diameter including the capsule measured on portal venous phase (PVP) images); (2) tumor location (left, junction, right, or caudate lobe); (3) tumor number (≤ 3 or > 3) [1]; (4) tumor shape (circular or irregular); (5) tumor margin (smooth or non-smooth); (6) intratumor necrosis (defined as nonenhancing areas with an intensity similar to that of gallbladder contents); (7) intratumor hemorrhage (defined as heterogeneous hyperintensity on T1-weighted (T1W) images and hypointensity on T2-weighted (T2W) images); (8) intratumor fat (defined as signal intensity drop on opposed-phase T1W images compared to in-phase images); (9) tumor encapsulation (defined as a peripheral rim of hyperenhancement on PVP or delayed phase (DP) images) [2]; (10) arterial peritumoral enhancement (defined as detectable enhancing portions adjacent to the tumor border on arterial phase (AP) images, later becoming isointensity compared with the background liver parenchyma on DP images) [3]; (11) satellite nodule (defined as small tumor nodule that was separated from the primary tumor with a similar type of enhancement, and had a tumor diameter and distance from the main tumor of no more than 2 cm) [4]; (12) arterial phase hyperenhancement (defined as whole or parts of the tumor with higher intensity than the rest of the liver on AP images); (13) washout appearance (defined as decreased enhancement of the tumor or part of it, compared to the liver parenchyma surrounding the tumor on PVP or DP images); (14) liver cirrhosis (defined as surface irregularity and nodularity, shrunken of liver, accompanied by ascites or signs of portal hypertension).

**S3. Detailed description of TACE procedure**

All conventional TACE procedures were carried out by two interventional radiologists with more than 10 and 5 years of experience with TACE. The Seldinger technique was used to puncture the right femoral artery, and a 5F catheter (RH catheter, Terumo, Tokyo, Japan) was inserted for celiac and hepatic artery angiography in evaluation of the vascular anatomy and tumor burden. For HCC patients who received TACE during February 2008 and June 2015, after superselective catheterization of hepatic artery feeding the tumor, an emulsion mixture of doxorubicin, hydroxycamptothecin, and Lipiodol was injected using a 2.7F microcatheter (Progreat microcatheter, Hengrui Pharmaceutical Co. Ltd., Lianyungang, China), followed by embolization of the tumor-feeding artery using gelatin sponge particles (1-2 mm). For HCC patients who received TACE during July 2015 and November 2019, after superselective catheterization of hepatic artery feeding the tumor, an emulsion mixture of lobaplatin, raltitrexed, and Lipiodol was injected using a 2.7F microcatheter, followed by embolization of the tumor-feeding artery using blank microsphere. The TACE procedure was stopped when tumor staining completely disappeared and regional arterial blood flow stopped.

Between February 2008 and June 2015, the patients for CR, PR, SD, and PD were 13 (20.0%), 17 (26.2%), 29 (44.6%), and 6 (9.2%), respectively. Between July 2015 and November 2019, the patients for CR, PR, SD, and PD were 14 (24.6%), 19 (33.3%), 20 (35.1%), and 4 (7.0%), respectively. The objective response rate between the two groups was not significantly different (46.2% vs. 57.9%, *F* = 1.676, *P* = 0.195).

**S4. Detailed name and description of radiomics features**

**4.1 Histogram features**

Histogram parameters are concerned with properties of individual pixels. They describe the distribution of voxel intensities within the MR image through commonly used and basic metrics. Let $X$ denote the three dimensional image matrix with $N$ voxels, and let $P$ denote the first order histogram divided by $N_{l}$ discrete intensity levels.

**1) MinIntensity:** The minimum intensity value of $X$.

**2) MaxIntensity:** The maximum intensity value of $X$.

**3) MedianIntensity:** The median intensity value of $X$.

## **4) Mean:**


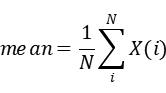


1. **StandardDeviation:**


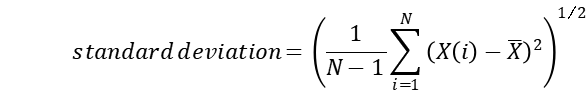


Where
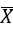
 is the mean of
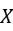
.

## **6) MeanDeviation:** The mean of the absolute deviations of all voxel intensities around the mean intensity value.

## **7) RelativeDeviation:** Let $\overline{X}$ denote the mean of a set of quantities $X_{i}$, then the relative deviation is defined by:


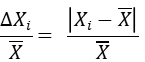


## **8) Variance:**


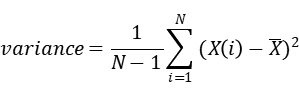

Where $\overline{X}$ is the mean of $X$.

## **9) Range:** The range of intensity values of $X$.

## **10) VolumeCount:** Describe the size of the ROI.

## **11) VoxelValueSum:** Represents the Sum calculations for voxels in the ROI.

## **12) RootMeanSquare (RMS):**


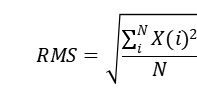


## **13) Skewness:**


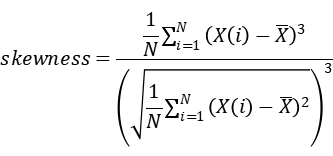

Where
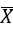
 is the mean of
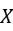
.

**14) Kurtosis:**


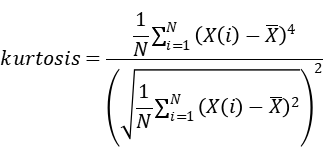

Where $\overline{X}$ is the mean of $X$.

## **15) Uniformity:**


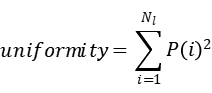


## **16) Energy:**


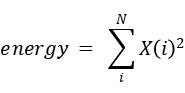


**17) Entropy:**


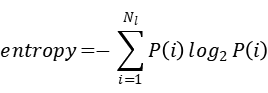


## **18) FrequencySize:** The ratio of the frequency of each object to the total frequency.

## **19) Percentile:** The percentile (p%) is defined as that value of the brightness $a$:


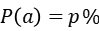


or equivalently:


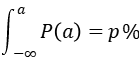


The P-th percentile of a list of N ordered values (sorted from least to greatest) is the smallest value in the list such that P percent of the data is less than or equal to that value. This is obtained by first calculating the ordinal rank and then taking the value from the ordered list that corresponds to that rank. The ordinal rank n is calculated using this formula:

*
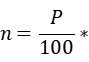
*

The 19 Percentiles are extracted: Percentile5, Percentile10, Percentile15, Percentile20, Percentile25, Percentile30, Percentile35, Percentile40, Percentile45, Percentile50, Percentile55, Percentile60, Percentile65, Percentile70, Percentile75, Percentile80, Percentile85, Percentile90, Percentile95.

**20) Quantile:** For a finite population of *N* equally probable values indexed 1, …, *N* from lowest to highest, the k-th q-quantile of this population can equivalently be computed via the value of:


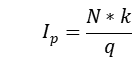


The 5 Quantiles are extracted: Quantile0.025, Quantile0.25, Quantile0.5, Quantile0.75, Quantile0.975.

**4.2 Grey level co-occurrence matrix (GLCM) features**

The grey level co-occurrence matrix (GLCM)
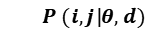
 represents the joint probability of certain sets of pixels having certain grey-level values. It calculates how many times a pixel with grey-level i occurs jointly with another pixel having a grey value j. By varying the displacement vector d between each pair of pixels.

The rotation angle of an offset: 0°, 45°, 90°, 135° and displacement vectors (distance to the neighbor pixel: 1, 2, 3 ...), different co-occurrence distributions from the same image of reference. GLCM of an image is computed using displacement vector d defined by its radius, (distance or count to the next adjacent neighbor preferably is equal to one) and rotational angles.

### GLCMEnergy:

### Formula:


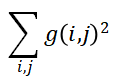


g is a GLCM

Where i,j are the spatial coordinates of g (i,j).

*Angle=All Direction, 0, 45, 90, 135, All_SD;

*Offset=1, 4, 7

The 18 features of GLCMEnergy are extracted:

GLCMEnergy_AllDirection_offset1, GLCMEnergy_AllDirection_offset1_SD, GLCMEnergy_angle0_offset1, GLCMEnergy_angle45_offset1,

GLCMEnergy_angle90_offset1, GLCMEnergy_angle135_offset1,

GLCMEnergy_AllDirection_offset4, GLCMEnergy_AllDirection_offset4_SD, GLCMEnergy_angle0_offset4, GLCMEnergy_angle45_offset4,

GLCMEnergy_angle90_offset4, GLCMEnergy_angle135_offset4,

GLCMEnergy_AllDirection_offset7, GLCMEnergy_angle0_offset7, GLCMEnergy_angle45_offset7, GLCMEnergy_angle90_offset7, GLCMEnergy_angle135_offset7, GLCMEnergy_AllDirection_offset7_SD

### 2) GLCMEntropy:

Formula:


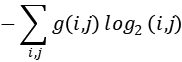


*Angle=All Direction, 0, 45, 90, 135, All_SD;

*Offset=1, 4, 7

The 18 features of GLCMEntropy are extracted:

GLCMEntropy_AllDirection_offset1, GLCMEntropy_AllDirection_offset1_SD, GLCMEntropy_angle0_offset1, GLCMEntropy_angle45_offset1, GLCMEntropy_angle90_offset1, GLCMEntropy_angle135_offset1,

GLCMEntropy_AllDirection_offset4, GLCMEntropy_AllDirection_offset4_SD, GLCMEntropy_angle0_offset4, GLCMEntropy_angle45_offset4, GLCMEntropy_angle90_offset4, GLCMEntropy_angle135_offset4,

GLCMEntropy_AllDirection_offset7, GLCMEntropy_AllDirection_offset7_SD, GLCMEntropy_angle0_offset7, GLCMEntropy_angle45_offset7, GLCMEntropy_angle90_offset7, GLCMEntropy_angle135_offset7

### 3) GLCMInertia:

Formula:


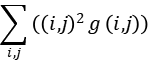


*Angle=All Direction, 0, 45, 90, 135, All_SD;

*Offset=1, 4, 7

The 18 features of GLCMInertia are extracted:

Inertia_AllDirection_offset1, Inertia _AllDirection_offset1_SD,

Inertia _angle0_offset1, Inertia _angle45_offset1,

Inertia _angle90_offset1, Inertia _angle135_offset1,

Inertia _AllDirection_offset4, Inertia _AllDirection_offset4_SD,

Inertia _angle0_offset4, Inertia _angle45_offset4,

Inertia _angle90_offset4, Inertia _angle135_offset4,

Inertia _AllDirection_offset7, Inertia _AllDirection_offset7_SD,

Inertia _angle0_offset7, Inertia _angle45_offset7,

Inertia _angle90_offset7, Inertia _angle135_offset7

### 4) Correlation:

Formula:

*
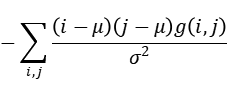
*

*Angle=All Direction, 0, 45, 90, 135, All_SD;

*Offset=1, 4, 7

The 18 features of Correlation are extracted:

Correlation_AllDirection_offset1, Correlation _AllDirection_offset1_SD,

Correlation _angle0_offset1, Correlation _angle45_offset1,

Correlation _angle90_offset1, Correlation _angle135_offset1,

Correlation _AllDirection_offset4, Correlation _AllDirection_offset4_SD,

Correlation _angle0_offset4, Correlation _angle45_offset4,

Correlation _angle90_offset4, Correlation _angle135_offset4,

Correlation _AllDirection_offset7, Correlation _AllDirection_offset7_SD,

Correlation _angle0_offset7, Correlation _angle45_offset7,

Correlation _angle90_offset7, Correlation _angle135_offset7

### 5) InverseDifferenceMoment:

Formula:


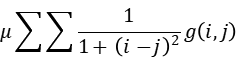


*Angle=All Direction, 0, 45, 90, 135, All_SD;

*Offset=1, 4, 7

The 18 features of InverseDifferenceMoment are extracted:

InverseDifferenceMoment_AllDirection_offset1, InverseDifferenceMoment_AllDirection_offset1_SD,

InverseDifferenceMoment_angle0_offset1,

InverseDifferenceMoment_angle45_offset1,

InverseDifferenceMoment_angle90_offset1,

InverseDifferenceMoment_angle135_offset1,

InverseDifferenceMoment_AllDirection_offset4,

InverseDifferenceMoment_AllDirection_offset4_SD,

InverseDifferenceMoment_angle0_offset4,

InverseDifferenceMoment_angle45_offset4,

InverseDifferenceMoment_angle90_offset4,

InverseDifferenceMoment_angle135_offset4,

InverseDifferenceMoment_AllDirection_offset7,

InverseDifferenceMoment_AllDirection_offset7_SD,

InverseDifferenceMoment_angle0_offset7,

InverseDifferenceMoment_angle45_offset7,

InverseDifferenceMoment_angle90_offset7,

InverseDifferenceMoment_angle135_offset7

### 6) ClusterShade:

Formula:


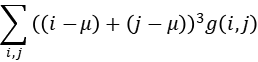


*Angle=All Direction, 0, 45, 90, 135, All_SD;

*Offset=1, 4, 7

The 18 features of ClusterShade are extracted:

ClusterShade_AllDirection_offset1, ClusterShade_AllDirection_offset1_SD,

ClusterShade_angle0_offset1, ClusterShade_angle45_offset1,

ClusterShade_angle90_offset1, ClusterShade_angle135_offset1,

ClusterShade_AllDirection_offset4, ClusterShade_AllDirection_offset4_SD,

ClusterShade_angle0_offset4, ClusterShade_angle45_offset4,

ClusterShade_angle90_offset4, ClusterShade_angle135_offset4,

ClusterShade_AllDirection_offset7, ClusterShade_AllDirection_offset7_SD,

ClusterShade_angle0_offset7, ClusterShade_angle45_offset7,

ClusterShade_angle90_offset7, ClusterShade_angle135_offset7

### 7) ClusterProminence:

Formula:


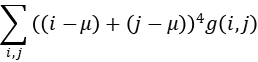


*Angle=All Direction, 0, 45, 90, 135, All_SD;

*Offset=1, 4, 7

The 18 features of ClusterProminence are extracted:

ClusterProminence_AllDirection_offset1, ClusterProminence_AllDirection_offset1_SD,

ClusterProminence_angle0_offset1, ClusterProminence_angle45_offset1,

ClusterProminence_angle90_offset1, ClusterProminence_angle135_offset1,

ClusterProminence_AllDirection_offset4, ClusterProminence_AllDirection_offset4_SD,

ClusterProminence_angle0_offset4, ClusterProminence_angle45_offset4,

ClusterProminence_angle90_offset4, ClusterProminence_angle135_offset4,

ClusterProminence_AllDirection_offset7, ClusterProminence_AllDirection_offset7_SD,

ClusterProminence_angle0_offset7, ClusterProminence_angle45_offset7,

ClusterProminence_angle90_offset7, ClusterProminence_angle135_offset7

### 8) HaralickCorrelation:

Formula:


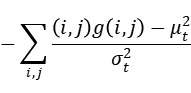


where $\mu_{t}$ and $\sigma_{t}$ are the mean and standard deviation of the row (or column, due to

symmetry) sums.

*Angle=All Direction, 0, 45, 90, 135, All_SD;

*Offset=1, 4, 7

The 18 features of HaralickCorrelation are extracted:

HaralickCorrelation_AllDirection_offset1, HaralickCorrelation_AllDirection_offset1_SD,

HaralickCorrelation_angle0_offset1, HaralickCorrelation_angle45_offset1,

HaralickCorrelation_angle90_offset1, HaralickCorrelation_angle135_offset1,

HaralickCorrelation_AllDirection_offset4, HaralickCorrelation_AllDirection_offset4_SD,

HaralickCorrelation_angle0_offset4, HaralickCorrelation_angle45_offset4,

HaralickCorrelation_angle90_offset4, HaralickCorrelation_angle135_offset4,

HaralickCorrelation_AllDirection_offset7, HaralickCorrelation_AllDirection_offset7_SD,

HaralickCorrelation_angle0_offset7, HaralickCorrelation_angle45_offset7,

HaralickCorrelation_angle90_offset7, HaralickCorrelation_angle135_offset7

**4.3 Grey level run length matrix (GLRLM) features**

The grey level run-length matrix (RLM) **P_r_ (i**, **j | θ**) is defined as the numbers of runs with pixels of grey level *i* and run length *j* for a given direction θ. RLMs is generated for each sample image segment having directions (0°,45°,90° &135°), then the following ten statistical features were derived.

1. **ShortRunEmphasis：**

$$SRE(\theta)=\frac{1}{n_{r}}\sum_{i=1}^{M} \sum_{j=1}^{N} \frac{p(i,j,\theta)}{j^{2}}$$

*Angle=All Direction, 0, 45, 90, 135, All_SD;

*Offset=1, 4, 7

The 18 features of ShortRunEmphasis are extracted:

ShortRunEmphasis_AllDirection_offset1, ShortRunEmphasis_AllDirection_offset1_SD,

ShortRunEmphasis_angle0_offset1, ShortRunEmphasis_angle45_offset1,

ShortRunEmphasis_angle90_offset1, ShortRunEmphasis_angle135_offset1,

ShortRunEmphasis_AllDirection_offset4, ShortRunEmphasis_AllDirection_offset4_SD,

ShortRunEmphasis_angle0_offset4, ShortRunEmphasis_angle45_offset4,

ShortRunEmphasis_angle90_offset4, ShortRunEmphasis_angle135_offset4,

ShortRunEmphasis_AllDirection_offset7, ShortRunEmphasis_AllDirection_offset7_SD,

ShortRunEmphasis_angle0_offset7, ShortRunEmphasis_angle45_offset7,

ShortRunEmphasis_angle90_offset7, ShortRunEmphasis_angle135_offset7

1. **LongRunEmphasis：**

$$LRE(\theta)=\frac{1}{n_{r}}\sum_{i=1}^{M} \sum_{j=1}^{N} p(i,j,\theta)j^{2}$$

*Angle=All Direction, 0, 45, 90, 135, All_SD;

*Offset=1, 4, 7

The 18 features of LongRunEmphasis are extracted:

LongRunEmphasis_AllDirection_offset1, LongRunEmphasis_AllDirection_offset1_SD,

LongRunEmphasis_angle0_offset1, LongRunEmphasis_angle45_offset1,

LongRunEmphasis_angle90_offset1, LongRunEmphasis_angle135_offset1,

LongRunEmphasis_AllDirection_offset4, LongRunEmphasis_AllDirection_offset4_SD,

LongRunEmphasis_angle0_offset4, LongRunEmphasis_angle45_offset4,

LongRunEmphasis_angle90_offset4, LongRunEmphasis_angle135_offset4,

LongRunEmphasis_AllDirection_offset7, LongRunEmphasis_AllDirection_offset7_SD,

LongRunEmphasis_angle0_offset7, LongRunEmphasis_angle45_offset7,

LongRunEmphasis_angle90_offset7, LongRunEmphasis_angle135_offset7

1. **GreyLevelNonuniformity：**

$$GLN(\theta)=\frac{1}{n_{r}}\sum_{i=1}^{M} (\sum_{j=1}^{N} p(i,j,\theta))^{2}$$

*Angle=All Direction, 0, 45, 90, 135, All_SD;

*Offset=1, 4, 7

The 18 features of GreyLevelNonuniformity are extracted:

GreyLevelNonuniformity_AllDirection_offset1, GreyLevelNonuniformity_AllDirection_offset1_SD,

GreyLevelNonuniformity_angle0_offset1, GreyLevelNonuniformity_angle45_offset1,

GreyLevelNonuniformity_angle90_offset1, GreyLevelNonuniformity_angle135_offset1,

GreyLevelNonuniformity_AllDirection_offset4, GreyLevelNonuniformity_AllDirection_offset4_SD,

GreyLevelNonuniformity_angle0_offset4, GreyLevelNonuniformity_angle45_offset4,

GreyLevelNonuniformity_angle90_offset4, GreyLevelNonuniformity_angle135_offset4,

GreyLevelNonuniformity_AllDirection_offset7, GreyLevelNonuniformity_AllDirection_offset7_SD,

GreyLevelNonuniformity_angle0_offset7, GreyLevelNonuniformity_angle45_offset7,

GreyLevelNonuniformity_angle90_offset7, GreyLevelNonuniformity_angle135_offset7

1. **RunLengthNonuniformity：**

$$RLN(\theta)=\frac{1}{n_{r}}\sum_{j=i}^{N} (\sum_{i=1}^{M} p(i,j,\theta))^{2}$$

*Angle=All Direction, 0, 45, 90, 135, All_SD;

*Offset=1, 4, 7

The 18 features of RunLengthNonuniformity are extracted:

RunLengthNonuniformity_AllDirection_offset1, RunLengthNonuniformity_AllDirection_offset1_SD,

RunLengthNonuniformity_angle0_offset1, RunLengthNonuniformity_angle45_offset1,

RunLengthNonuniformity_angle90_offset1, RunLengthNonuniformity_angle135_offset1,

RunLengthNonuniformity_AllDirection_offset4, RunLengthNonuniformity_AllDirection_offset4_SD,

RunLengthNonuniformity_angle0_offset4, RunLengthNonuniformity_angle45_offset4,

RunLengthNonuniformity_angle90_offset4, RunLengthNonuniformity_angle135_offset4,

RunLengthNonuniformity_AllDirection_offset7, RunLengthNonuniformity_AllDirection_offset7_SD,

RunLengthNonuniformity_angle0_offset7, RunLengthNonuniformity_angle45_offset7,

RunLengthNonuniformity_angle90_offset7, RunLengthNonuniformity_angle135_offset7

1. **LowGreyLevelRunEmphasis：**

$$LGRE(\theta)=\frac{1}{n_{r}}\sum_{j=i}^{N} \sum_{i=1}^{M} \frac{p(i,j,\theta)}{i^{2}}$$

*Angle=All Direction, 0, 45, 90, 135, All_SD;

*Offset=1, 4, 7

The 18 features of LowGreyLevelRunEmphasis are extracted:

LowGreyLevelRunEmphasis_AllDirection_offset1, LowGreyLevelRunEmphasis_AllDirection_offset1_SD,

LowGreyLevelRunEmphasis_angle0_offset1, LowGreyLevelRunEmphasis_angle45_offset1,

LowGreyLevelRunEmphasis_angle90_offset1, LowGreyLevelRunEmphasis_angle135_offset1,

LowGreyLevelRunEmphasis_AllDirection_offset4, LowGreyLevelRunEmphasis_AllDirection_offset4_SD,

LowGreyLevelRunEmphasis_angle0_offset4, LowGreyLevelRunEmphasis_angle45_offset4,

LowGreyLevelRunEmphasis_angle90_offset4, LowGreyLevelRunEmphasis_angle135_offset4,

LowGreyLevelRunEmphasis_AllDirection_offset7, LowGreyLevelRunEmphasis_AllDirection_offset7_SD,

LowGreyLevelRunEmphasis_angle0_offset7, LowGreyLevelRunEmphasis_angle45_offset7,

LowGreyLevelRunEmphasis_angle90_offset7, LowGreyLevelRunEmphasis_angle135_offset7

1. **HighGreyLevelRunEmphasis：**

$$HGRE(\theta)=\frac{1}{n_{r}}\sum_{j=i}^{N} \sum_{i=1}^{M} p(i,j,\theta)i^{2}$$

*Angle=All Direction, 0, 45, 90, 135, All_SD;

*Offset=1, 4, 7

The 18 features of HighGreyLevelRunEmphasis are extracted:

HighGreyLevelRunEmphasis_AllDirection_offset1, HighGreyLevelRunEmphasis_AllDirection_offset1_SD,

HighGreyLevelRunEmphasis_angle0_offset1, HighGreyLevelRunEmphasis_angle45_offset1,

HighGreyLevelRunEmphasis_angle90_offset1, HighGreyLevelRunEmphasis_angle135_offset1,

HighGreyLevelRunEmphasis_AllDirection_offset4, HighGreyLevelRunEmphasis_AllDirection_offset4_SD,

HighGreyLevelRunEmphasis_angle0_offset4, HighGreyLevelRunEmphasis_angle45_offset4,

HighGreyLevelRunEmphasis_angle90_offset4, HighGreyLevelRunEmphasis_angle135_offset4,

HighGreyLevelRunEmphasis_AllDirection_offset7, HighGreyLevelRunEmphasis_AllDirection_offset7_SD,

HighGreyLevelRunEmphasis_angle0_offset7, HighGreyLevelRunEmphasis_angle45_offset7,

HighGreyLevelRunEmphasis_angle90_offset7, HighGreyLevelRunEmphasis_angle135_offset7

1. **ShortRunLowGreyLevelEmphasis：**

$$SRLGE(\theta)=\frac{1}{n_{r}}\sum_{j=i}^{N} \sum_{i=1}^{M} \frac{p(i,j,\theta)}{i^{2}j^{2}}$$

*Angle=All Direction, 0, 45, 90, 135, All_SD;

*Offset=1, 4, 7

The 18 features of ShortRunLowGreyLevelEmphasis are extracted:

ShortRunLowGreyLevelEmphasis_AllDirection_offset1, ShortRunLowGreyLevelEmphasis_AllDirection_offset1_SD,

ShortRunLowGreyLevelEmphasis_angle0_offset1, ShortRunLowGreyLevelEmphasis_angle45_offset1,

ShortRunLowGreyLevelEmphasis_angle90_offset1, ShortRunLowGreyLevelEmphasis_angle135_offset1,

ShortRunLowGreyLevelEmphasis_AllDirection_offset4, ShortRunLowGreyLevelEmphasis_AllDirection_offset4_SD,

ShortRunLowGreyLevelEmphasis_angle0_offset4, ShortRunLowGreyLevelEmphasis_angle45_offset4,

ShortRunLowGreyLevelEmphasis_angle90_offset4, ShortRunLowGreyLevelEmphasis_angle135_offset4,

ShortRunLowGreyLevelEmphasis_AllDirection_offset7, ShortRunLowGreyLevelEmphasis_AllDirection_offset7_SD,

ShortRunLowGreyLevelEmphasis_angle0_offset7, ShortRunLowGreyLevelEmphasis_angle45_offset7,

ShortRunLowGreyLevelEmphasis_angle90_offset7, ShortRunLowGreyLevelEmphasis_angle135_offset7

1. **ShortRunHighGreyLevelEmphasis：**

$$SRHGE(\theta)=\frac{1}{n_{r}}\sum_{j=i}^{N} \sum_{i=1}^{M} \frac{p(i,j,\theta)i^{2}}{j^{2}}$$

*Angle=All Direction, 0, 45, 90, 135, All_SD;

*Offset=1, 4, 7

The 18 features of ShortRunHighGreyLevelEmphasis are extracted:

ShortRunHighGreyLevelEmphasis_AllDirection_offset1, ShortRunHighGreyLevelEmphasis_AllDirection_offset1_SD,

ShortRunHighGreyLevelEmphasis_angle0_offset1, ShortRunHighGreyLevelEmphasis_angle45_offset1,

ShortRunHighGreyLevelEmphasis_angle90_offset1, ShortRunHighGreyLevelEmphasis_angle135_offset1,

ShortRunHighGreyLevelEmphasis_AllDirection_offset4, ShortRunHighGreyLevelEmphasis_AllDirection_offset4_SD,

ShortRunHighGreyLevelEmphasis_angle0_offset4, ShortRunHighGreyLevelEmphasis_angle45_offset4,

ShortRunHighGreyLevelEmphasis_angle90_offset4, ShortRunHighGreyLevelEmphasis_angle135_offset4,

ShortRunHighGreyLevelEmphasis_AllDirection_offset7, ShortRunHighGreyLevelEmphasis_AllDirection_offset7_SD,

ShortRunHighGreyLevelEmphasis_angle0_offset7, ShortRunHighGreyLevelEmphasis_angle45_offset7,

ShortRunHighGreyLevelEmphasis_angle90_offset7, ShortRunHighGreyLevelEmphasis_angle135_offset7

1. **LongRunLowGreyLevelEmphasis:**

$$LRLGE(\theta)=\frac{1}{n_{r}}\sum_{j=i}^{N} \sum_{i=1}^{M} \frac{p(i,j,\theta)j^{2}}{i^{2}}$$

*Angle=All Direction, 0, 45, 90, 135, All_SD;

*Offset=1, 4, 7

The 18 features of LongRunLowGreyLevelEmphasis are extracted:

LongRunLowGreyLevelEmphasis_AllDirection_offset1, LongRunLowGreyLevelEmphasis_AllDirection_offset1_SD,

LongRunLowGreyLevelEmphasis_angle0_offset1, LongRunLowGreyLevelEmphasis_angle45_offset1,

LongRunLowGreyLevelEmphasis_angle90_offset1, LongRunLowGreyLevelEmphasis_angle135_offset1,

LongRunLowGreyLevelEmphasis_AllDirection_offset4, LongRunLowGreyLevelEmphasis_AllDirection_offset4_SD,

LongRunLowGreyLevelEmphasis_angle0_offset4, LongRunLowGreyLevelEmphasis_angle45_offset4,

LongRunLowGreyLevelEmphasis_angle90_offset4, LongRunLowGreyLevelEmphasis_angle135_offset4,

LongRunLowGreyLevelEmphasis_AllDirection_offset7, LongRunLowGreyLevelEmphasis_AllDirection_offset7_SD,

LongRunLowGreyLevelEmphasis_angle0_offset7, LongRunLowGreyLevelEmphasis_angle45_offset7,

LongRunLowGreyLevelEmphasis_angle90_offset7, LongRunLowGreyLevelEmphasis_angle135_offset7

1. **LongRunHighGreyLevelEmphasis:**

$$LRHGE(\theta)=\frac{1}{n_{r}}\sum_{j=i}^{N} \sum_{i=1}^{M} p(i,j,\theta)i^{2}j^{2}$$

where *n_r_* is the total number of runs and *n_p_* is the number of pixels in the image.

*Angle=All Direction, 0, 45, 90, 135, All_SD;

*Offset=1, 4, 7

The 18 features of LongRunHighGreyLevelEmphasis are extracted:

LongRunHighGreyLevelEmphasis_AllDirection_offset1, LongRunHighGreyLevelEmphasis_AllDirection_offset1_SD,

LongRunHighGreyLevelEmphasis_angle0_offset1, LongRunHighGreyLevelEmphasis_angle45_offset1,

LongRunHighGreyLevelEmphasis_angle90_offset1, LongRunHighGreyLevelEmphasis_angle135_offset1,

LongRunHighGreyLevelEmphasis_AllDirection_offset4, LongRunHighGreyLevelEmphasis_AllDirection_offset4_SD,

LongRunHighGreyLevelEmphasis_angle0_offset4, LongRunHighGreyLevelEmphasis_angle45_offset4,

LongRunHighGreyLevelEmphasis_angle90_offset4, LongRunHighGreyLevelEmphasis_angle135_offset4,

LongRunHighGreyLevelEmphasis_AllDirection_offset7, LongRunHighGreyLevelEmphasis_AllDirection_offset7_SD,

LongRunHighGreyLevelEmphasis_angle0_offset7, LongRunHighGreyLevelEmphasis_angle45_offset7,

LongRunHighGreyLevelEmphasis_angle90_offset7, LongRunHighGreyLevelEmphasis_angle135_offset7

**4.4 Grey-level zone size matrix (GLZSM) features**

The grey level Size Zone Matrix (SZM) is the starting point of Thibault matrices. For a texture image f with N grey levels, it is denoted GSf(s, g) and provides a statistical representation by the estimation of a bivariate conditional probability density function of the image distribution values. It is calculated according to the pioneering Run Length Matrix principle: the value of the matrix GSf(s, g) is equal to the number of zones of size s and of grey level g. The resulting matrix has a fixed number of lines equal to N, the number of grey levels, and a dynamic number of columns, determined by the size of the largest zone as well as the size quantization.

The more homogeneous the texture, the wider and flatter the matrix. SZM does not required computation in several directions. However, it has been empirically proved that the degree of grey level quantization still has an important impact on the texture classification performance.

Let P define the GLSZM of a quantized volume V (x,y,z) with isotropic voxel size. *P(i,j)* represents the number of 3D zones of grey-levels *i* and of size j in V , *Ng* represents the pre-defined number of quantized grey-levels set in V, and *Lz* represents the size of the largest zone (of any grey-level) in V. One GLSZM of size *Ng*× *Lz* is computed per volume V by adding up all possible largest zone-sizes, with zones constructed from 26-connected neighbours of the same grey-level in 3D space (one voxel can be part of only one zone). The entry *(i,j)* of the normalized GLSZM is then defined as:


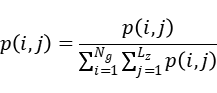


The following quantities are also defined:

*
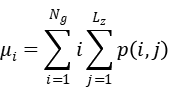
*


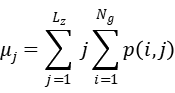


## **1) SmallAreaEmphasis:**


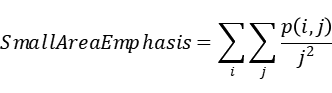


## **2) LargeAreaEmphasis:**


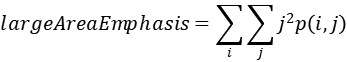


## **3) IntensityVariability:**


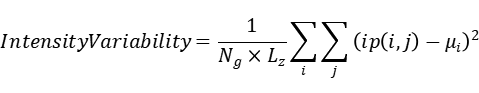


## **4) SizeZoneVariability:**


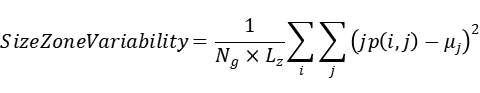


## **5) ZonePercentage**


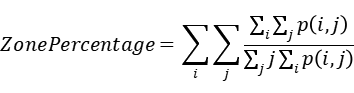


## **6) LowIntensityEmphasis:**


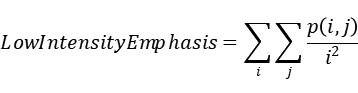


## **7) HighIntensityEmphasis:**


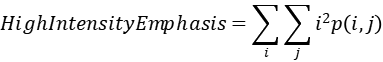


## **8) LowIntensitySmallAreaEmphasis:**


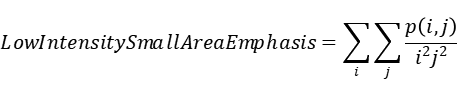


## **9) HighIntensitySmallAreaEmphasis:**


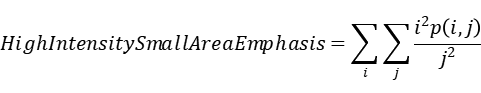


**10) LowIntensityLargeAreaEmphasis:**

**
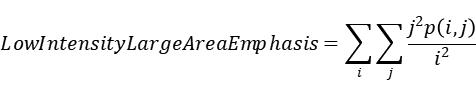
**

## **HighIntensityLargeAreaEmphasis:**


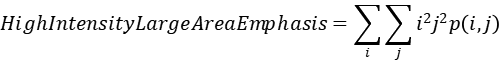


**4.5 Haralick features**

### 1) HaraEntropy:

Formula:
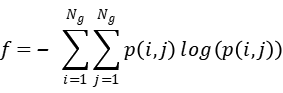


where $\mu_{t}$ and $\sigma_{t}$ are the mean and standard deviation of the row (or column, due to symmetry) sums.

### 2) Angular Second Moment:

Formula:


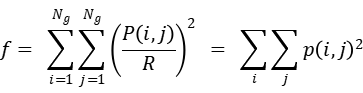


### 3) Contrast:

Formula:


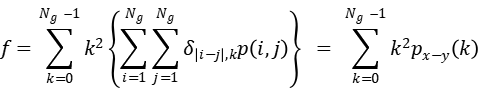


### 4) Haralick Variance:

Formula:


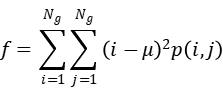


### 5) SumAverage:

Formula:

**
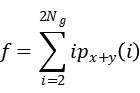
**

### 6) SumVariance:

Formula:
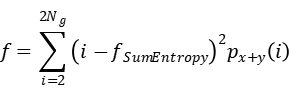


### 7) SumEntropy:

Formula:


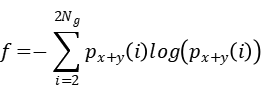


### 8) Difference Variance:

Formula:


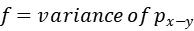


### 9) Difference Entropy:

Formula:

**
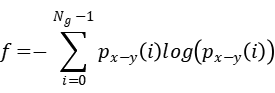
**

### 10) Inverse Difference Moment:

Formula:


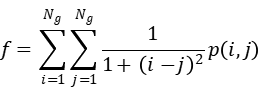


**4.6 Form factors**

These group of features includes descriptors of the three-dimensional size and shape of the tumor region. Let in the following definitions *V* denote the volume and *A* the surface area of the volume of interest. We determined the following shape and size based features:

**1) MeshVolume:**

Formula:

**
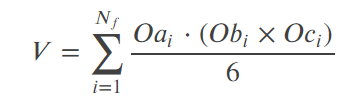
**

The volume of the ROI *V* is calculated from the triangle mesh of the ROI. For each face *i* in the mesh, defined by points *a_i_*, *b_i_* and *c_i_*, the (signed) volume *V_f_* of the tetrahedron defined by that face and the origin of the image (*O*) is calculated.

**2) OneVoxelVolume:** The volume of a single voxel.

**3) VoxelVolume:** The volume (*V*) of the tumor is determined by counting the number of pixels in the tumor region and multiplying this value by the voxel size.

**4) Surface Area:**


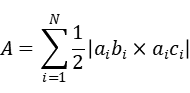


**5) Surface to Volume Ratio:**

**
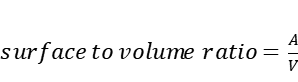
**

**6) Sphericity:**

**
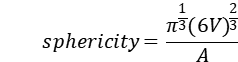
**

**7) Compactness 1:**

**
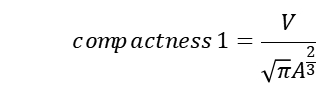
**

**8) Compactness 2:**

**
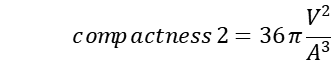
**

**9) Spherical Disproportion:**


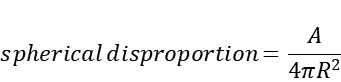


Where *R* is the radius of a sphere with the same volume as the tumor.

Where *N* is the total number of triangles covering the surface and *a*, *b* and *c* are edge vectors of the triangles.

**10) Maximum 3D diameter:** The maximum three-dimensional tumor diameter is measured as the largest pairwise Euclidean distance, between voxels on the surface of the tumor volume.

**11) MajorAxisLength:**

**
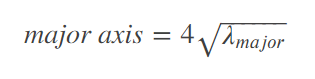
**

This feature yield the largest axis length of the ROI-enclosing ellipsoid and is calculated using the largest principal component *λ_major_*.

**12) MinorAxisLength:**

**
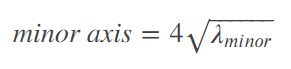
**

This feature yield the second-largest axis length of the ROI-enclosing ellipsoid and is calculated using the largest principal component *λ_minor_*.

**13) LeastAxisLength:**

**
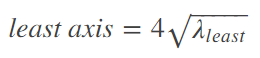
**

This feature yield the smallest axis length of the ROI-enclosing ellipsoid and is calculated using the largest principal component *λ_least_*.

**14) Elongation:**

**
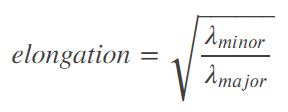
**

Here, *λ_major_* and *λ_minor_* are the lengths of the largest and second largest principal component axes.

**15) Flatness:**

**
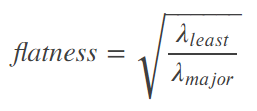
**

Here, *λ_major_* and *λ_least_* are the lengths of the largest and smallest principal component axes. The values range between 1 (non-flat, sphere-like) and 0 (a flat object, or single-slice segmentation).

**4.7 Gaussian transform features**

The method of image filter processing is Laplacian of Gaussian (LoG). LoG filter is an edge enhancement filter, which emphasizes areas of grey level change, where sigma defines how coarse the emphasized texture should be. A low sigma emphasis on fine textures (change over a short distance), where a high sigma value emphasizes coarse textures (grey level change over a large distance). One sigma value was used in this study (1 mm).

**S5. The formulae of the rad-score in each phase and their combination**

| Rad-score | Calculation formula |
| --- | --- |
| Rad-score_AP | = - 0.353 - 1.535 × AP_LoG_LongRunEmphasis_angle0_offset1 +  0.269 × AP_IntensityVariability +  0.335 × AP_LoG_Percentile90 +  0.102 × AP_GreyLevelNonuniformity_angle90_offset1 +  0.136 × AP_LoG_Variance +  0.031 × AP_VoxelValueSum -  0.201 × AP_GLCMEntropy_angle90_offset7 |
| Rad-score_PVP | = 0.002 + 0.537 × PVP_ClusterProminence_angle45_offset7 +  1.044 × PVP_GreyLevelNonuniformity_angle135_offset1 +  0.747 × PVP_HaralickCorrelation_angle45_offset7 +  0.509 × PVP_LoG_GreyLevelNonuniformity_angle45_offset7 -  0.458 × PVP_GLCMEntropy_angle135_offset4 -  0.890 × PVP_LeastAxisLength |
| Rad-score_DP | = 0.056 + 0.522 × DP_GreyLevelNonuniformity_AllDirection_offset4_SD -  0.290 × DP_GreyLevelNonuniformity_angle90_offset1 +  0.031 × DP_Compactness1 +  0.072 × DP_MaxIntensity +  0.349 × DP_ClusterProminence_angle135_offset1 +  0.772 × DP_LoG_GreyLevelNonuniformity_AllDirection_offset1_SD -  0.236 × DP_LoG_GreyLevelNonuniformity_angle45_offset7 +  0.168 × DP_LoG_sumAverage -  0.139 × DP_GLCMEntropy_angle135_offset7 +  0.478 × DP_VoxelValueSum |
| Rad-score_AP-PVP | = - 0.063 - 0.517 × AP_LoG_LongRunEmphasis_angle0_offset1 -  0.119 × PVP_ShortRunEmphasis_AllDirection_offset7_SD -  0.377 × PVP_GLCMEntropy_angle45_offset7 +  0.560 × PVP_GreyLevelNonuniformity_angle90_offset1 +  0.828 × PVP_HaralickCorrelation_angle45_offset7 +  0.345 × PVP_LoG_GreyLevelNonuniformity_angle135_offset7 |
| Rad-score_AP-DP | = - 0.161 - 0.989 × AP_LoG_LongRunEmphasis_angle0_offset1 +  0.408 × AP_ClusterProminence_angle45_offset7 -  0.136 × AP_GLCMEnergy_angle90_offset4 +  0.427 × DP_LoG_GreyLevelNonuniformity_angle45_offset7 -  0.605 × DP_GLCMEntropy_angle135_offset7 |
| Rad-score_PVP-DP | = 0.040 - 0.610 × PVP_GreyLevelNonuniformity_angle0_offset1 +  0.227 × PVP_ClusterProminence_angle45_offset7 +  1.389 × PVP_GreyLevelNonuniformity_angle135_offset1 +  0.925 × PVP_HaralickCorrelation_angle45_offset7 -  0.081 × PVP_GLCMEntropy_angle135_offset4 +  0.235 × DP_LoG_GreyLevelNonuniformity_angle45_offset7 -  0.593 × DP_GLCMEntropy_angle135_offset7 |
| Rad-score_AP-PVP-DP | = - 0.070 - 0.784 × AP_LoG_LongRunEmphasis_angle0_offset1 +  0.452 × AP_ClusterProminence_angle45_offset7 +  0.642 × PVP_GreyLevelNonuniformity_angle135_offset1 +  0.856 × PVP_HaralickCorrelation_angle45_offset7 +  0.225 × DP_LoG_GreyLevelNonuniformity_angle45_offset7 -  0.745 × DP_GLCMEntropy_angle135_offset7 |

**Note:** *Rad-score*, radiomics score; *AP*, arterial phase; *PVP*, portal venous phase; *DP*, delayed phase; *LoG*, laplacian of gaussian; *GLCM*, grey level co-occurrence matrix.

**S6. Univariate and multivariate analyses of clinical-radiological characteristics between objective response and non-response groups in the training cohort**

| Factors | Univariate analysis | | |  | Multivariate analysis | | |
| --- | --- | --- | --- | --- | --- | --- | --- |
|  | β | OR (95% CI) | *P* value |  | β | OR (95% CI) | *P* value |
| Intercept | — | — | — |  | 0.760 | 2.138 (0.812 - 5.626) | 0.124 |
| Age (years) | -0.553×10^-3^ | 0.999 (0.950 - 1.051) | 0.983 |  |  |  |  |
| Gender | 0.078 | 1.081 (0.252 - 4.638) | 0.916 |  |  |  |  |
| History of hepatitis B or C | -0.853 | 0.426 (0.170 - 1.069) | 0.069 |  | — | — | — |
| AFP (IU/ml) | -0.098 | 0.907 (0.372 - 2.212) | 0.831 |  |  |  |  |
| ALT (U/L) | 0.128 | 1.136 (0.483 - 2.675) | 0.770 |  |  |  |  |
| AST (U/L) | 0.515 | 1.673 (0.679 - 4.124) | 0.263 |  |  |  |  |
| GGT (U/L) | 0.548 | 1.730 (0.631 - 4.740) | 0.287 |  |  |  |  |
| ALP (U/L) | 0.610 | 1.841 (0.768 - 4.412) | 0.171 |  |  |  |  |
| TBIL (umol/L) | -0.949 | 0.387 (0.160 - 0.938) | 0.036* |  | -1.072 | 0.342 (0.130 - 0.904) | 0.031* |
| ALB (g/L) | 0.109 | 1.115 (0.458 - 2.717) | 0.810 |  |  |  |  |
| PLT (×10^9^/L) | 0.421 | 1.524 (0.647 - 3.586) | 0.335 |  |  |  |  |
| PT (s) | -0.276 | 0.759 (0.318 - 1.815) | 0.535 |  |  |  |  |
| Child-Pugh class | -0.170 | 0.844 (0.308 - 2.308) | 0.741 |  |  |  |  |
| ECOG performance status | 0.449 | 1.567 (0.551 - 4.458) | 0.400 |  |  |  |  |
| BCLC stage | 0.316 | 1.371 (0.758 - 2.481) | 0.297 |  |  |  |  |
| Tumor size (cm) | 1.130 | 3.088 (1.251 - 7.624) | 0.014* |  | — | — | — |
| Tumor location | 0.037 | 1.038 (0.446 - 2.414) | 0.931 |  |  |  |  |
| Tumor number | -0.485 | 0.616 (0.137 - 2.758) | 0.526 |  |  |  |  |
| Tumor shape | 1.650 | 5.185 (1.540 - 17.453) | 0.008* |  | 1.497 | 4.468 (1.216 - 16.415) | 0.024* |
| Tumor margin | 0.476 | 1.609 (0.595 - 4.351) | 0.348 |  |  |  |  |
| Intratumor necrosis | 0.457 | 1.579 (0.602 - 4.140) | 0.353 |  |  |  |  |
| Intratumor hemorrhage | 0.622 | 1.862 (0.672 - 5.162) | 0.232 |  |  |  |  |
| Intratumor fat | -0.722 | 0.486 (0.135 - 1.758) | 0.272 |  |  |  |  |
| Tumor encapsulation | -1.150 | 0.317 (0.127 - 0.794) | 0.014* |  | -1.039 | 0.354 (0.130 - 0.964) | 0.042* |
| Arterial peritumoral enhancement | 0.221 | 1.247 (0.465 - 3.345) | 0.662 |  |  |  |  |
| Satellite nodule | 2.180 | 8.853 (1.038 - 75.475) | 0.046* |  | — | — | — |
| Arterial phase hyperenhancement | -0.821 | 0.440 (0.076 - 2.545) | 0.360 |  |  |  |  |
| Washout appearance | -1.110 | 0.329 (0.126 - 0.856) | 0.023* |  | — | — | — |
| Liver cirrhosis | -0.423 | 0.655 (0.266 - 1.614) | 0.358 |  |  |  |  |

**Note:** Variables with *P* value < 0.1 in the univariate analysis were included in the multivariate logistic regression analysis (stepwise elimination), and β is the regression coefficient. * *P* value < 0.05. *AFP*, alpha-fetoprotein; *ALT*, alanine aminotransferase; *AST*, aspartate aminotransferase; *GGT*, γ-glutamyltranspeptadase; *ALP*, alkaline phosphatase; *TBIL*, total bilirubin; *ALB*, albumin; *PLT*, platelet count; *PT*, prothrombin time; *ECOG*, Eastern Cooperative Oncology Group; *BCLC*, Barcelona Clinic Liver Cancer.

**S7. Stratified prediction performance on the subgroups of MRI scanner**

| Model | AUC (95 %CI) | *P* value |
| --- | --- | --- |
| AP model |  | 0.435^a^ |
| 1.5 T | 0.777 (0.687 - 0.867) | 0.833^b^ |
| 3.0 T | 0.675 (0.441 - 0.910) | 0.493^c^ |
| Overall cohort | 0.764 (0.680 - 0.847) |  |
| PVP model |  | 0.878^a^ |
| 1.5 T | 0.796 (0.708 - 0.884) | 0.973^b^ |
| 3.0 T | 0.812 (0.630 - 0.994) | 0.859^c^ |
| Overall cohort | 0.794 (0.715 - 0.873) |  |
| DP model |  | 0.250^a^ |
| 1.5 T | 0.767 (0.674 - 0.860) | 0.737^b^ |
| 3.0 T | 0.590 (0.309 - 0.870) | 0.309^c^ |
| Overall cohort | 0.745 (0.657 - 0.833) |  |
| AP-PVP model |  | 0.958^a^ |
| 1.5 T | 0.815 (0.732 - 0.897) | 0.968^b^ |
| 3.0 T | 0.821 (0.628 - 1.000) | 0.940^c^ |
| Overall cohort | 0.813 (0.737 - 0.888) |  |
| AP-DP model |  | 0.853^a^ |
| 1.5 T | 0.786 (0.695 - 0.876) | 0.911^b^ |
| 3.0 T | 0.761 (0.516 - 1.000) | 0.893^c^ |
| Overall cohort | 0.779 (0.695 - 0.862) |  |
| PVP-DP model |  | 0.828^a^ |
| 1.5 T | 0.796 (0.709 - 0.883) | 0.978^b^ |
| 3.0 T | 0.769 (0.547 - 0.992) | 0.815^c^ |
| Overall cohort | 0.798 (0.719 - 0.877) |  |
| AP-PVP-DP model |  | 0.981^a^ |
| 1.5 T | 0.815 (0.732 - 0.898) | 0.984^b^ |
| 3.0 T | 0.812 (0.594 - 1.000) | 0.973^c^ |
| Overall cohort | 0.816 (0.740 - 0.892) |  |

**Note:** *AP*, arterial phase; *PVP*, portal venous phase; *DP*, delayed phase; AUC, area under the curve; *CI*, confidence interval.

^a^ *P* value: the AUC values between the subgroups of MRI scanner were calculated by Delong’s test. ^b^ *P* value: the AUC values between the 1.5 T subgroup and the overall cohort were calculated by Delong’s test. ^c^ *P* value: the AUC values between the 3.0 T subgroup and the overall cohort were calculated by Delong’s test.

**S8. Performance evaluation of the radiological-radiomics model**

The radiological-radiomics model which incorporated independent radiological predictors (tumor shape and tumor encapsulation) and radiomics score (based on AP-PVP-DP model) was constructed, showing favourable predictive performance with AUCs of 0.854 (95% CI, 0.775 - 0.933) and 0.848 (95% CI, 0.712 - 0.984) in the training and validation cohorts. The accuracy, sensitivity, specificity of the radiological-radiomics model were 0.776, 0.707, and 0.841 in the training cohort and 0.757, 0.889, and 0.632 in the validation cohort. In the both cohorts, the radiological-radiomics model demonstrated comparative performance compared with the combined model (training: AUC, 0.854 vs. 0.878, *P* = 0.259; validation: AUC, 0.848 vs. 0.833, *P* = 0.489). ROC curves of the radiological-radiomics model and combined model are shown in **Figure S1**.

**
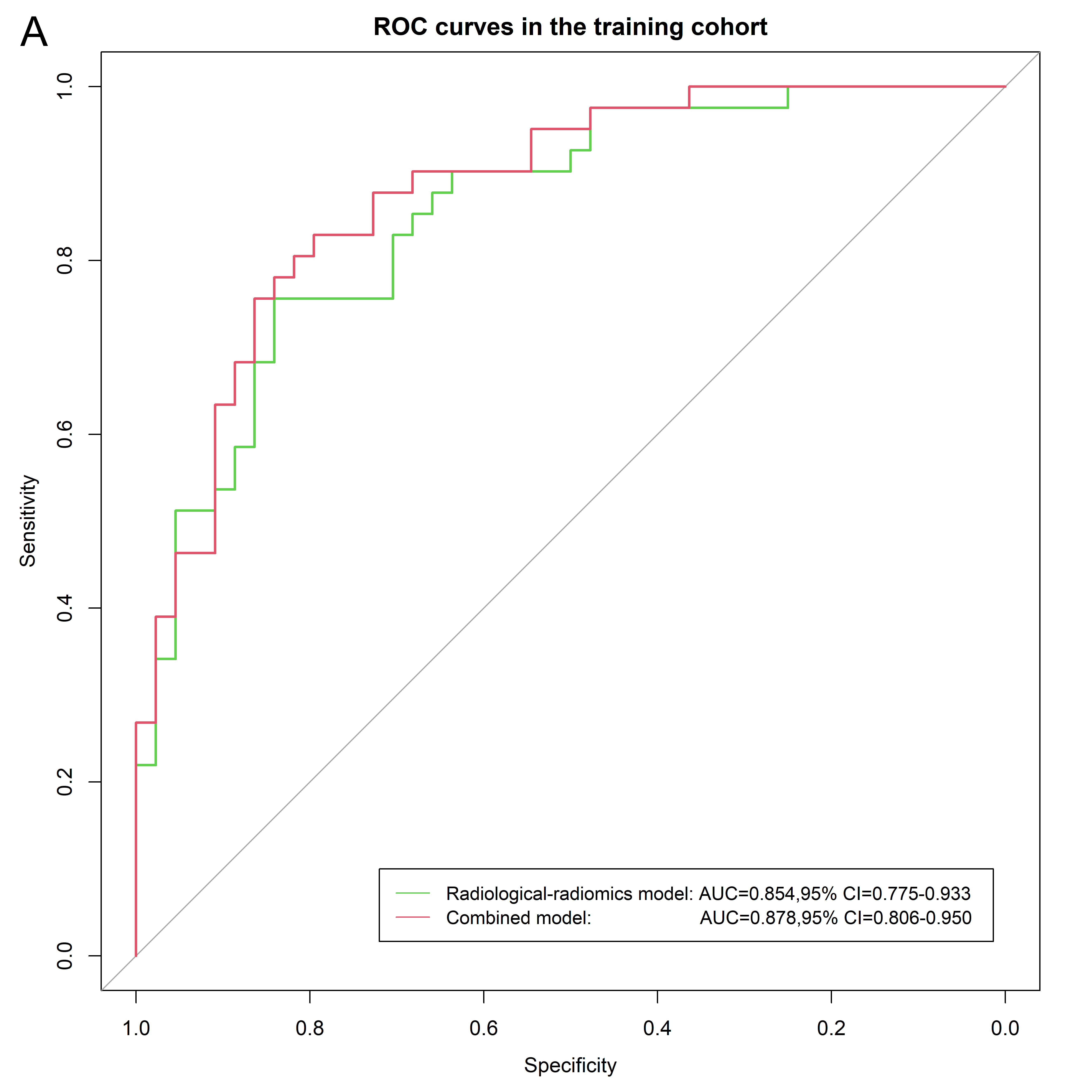

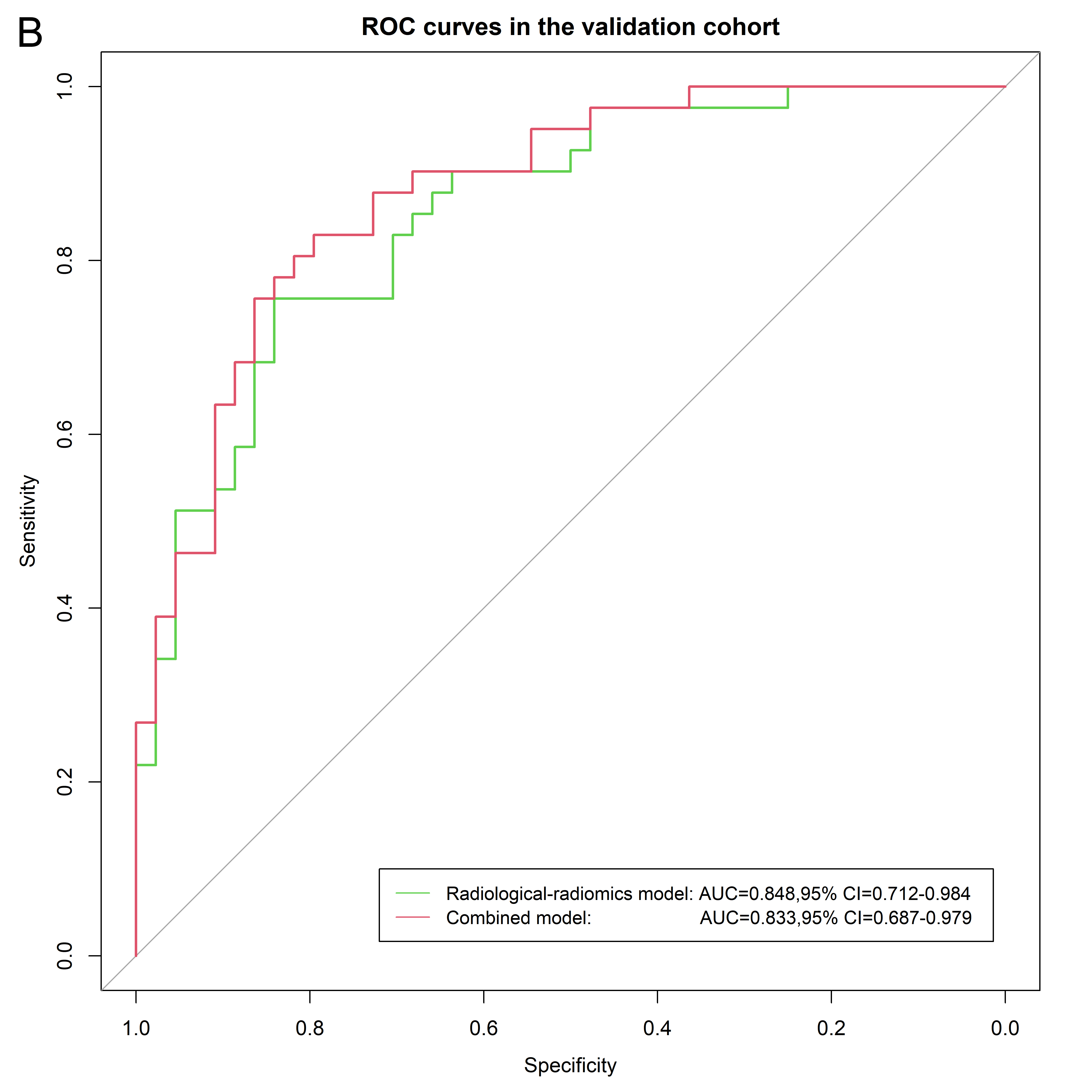
**

**Figure S1.** ROC curves for radiological-radiomics model and combined model in the training cohort **(A)** and validation cohort **(B)**.

**S9. References**

1. Kim BK, Kim SU, Kim KA, Chung YE, Kim MJ, Park MS, et al. Complete response at first chemoembolization is still the most robust predictor for favorable outcome in hepatocellular carcinoma. J Hepatol (2015) 62:1304-1310. doi: 10.1016/j.jhep.2015.01.022
2. Ronot M, Purcell Y, Vilgrain V. Hepatocellular Carcinoma: Current Imaging Modalities for Diagnosis and Prognosis. Dig Dis Sci. (2019) 64:934-950. doi: 10.1007/s10620-019-05547-0
3. Rosiak G, Podgórska J, Rosiak E, Cieszanowski A. CT/MRI LI-RADS v2017 - review of the guidelines. Pol J Radiol. (2018) 83:e355-e365. doi: 10.5114/pjr.2018.78391.
4. Song W, Yu X, Guo D, Liu H, Tang Z, Liu X, et al. MRI-Based Radiomics: Associations With the Recurrence-Free Survival of Patients With Hepatocellular Carcinoma Treated With Conventional Transcatheter Arterial Chemoembolization. J Magn Reson Imaging. (2020) 52:461-473. doi: 10.1002/jmri.26977
